# Impact of decoration of spherical silicon nanoparticles on 2D Ni-MOF nanosheets for integrating superior photodegradation of toxic organic pollutants in aqueous solution

**DOI:** 10.1038/s41598-025-17952-9

**Published:** 2025-09-12

**Authors:** Amin M. Elkony, Hosni A. Gomaa, Ahmed A. Omran, Nour F. Attia

**Affiliations:** 1 Department of Chemistry, Faculty of Science, Al-Azhar University, Nasr City, Cairo, 11884 Egypt; 2 Gas Analysis and Fire Safety Laboratory, Chemistry Division, National Institute for Standards, P. O. Box 136, Giza, 12211 Egypt

**Keywords:** 2D Ni–MOF nanosheets, Silicon nanoparticles, Photocatalytic degradation, Bandgap engineering, Malachite green, Tetracycline, Crystal violet, Chemistry, Environmental sciences, Materials science, Nanoscience and technology

## Abstract

The contamination of water resources by toxic organic pollutants poses severe environmental and health risks, representing a critical global challenge that demands the development of efficient, cost-effective, and sustainable remediation strategies. Herein, we report a scalable synthesis of a novel photocatalyst consisting of two-dimensional (2D) nanosheets of a nickel-based metal–organic framework (Ni-MOF) in conjunction with low-bandgap silicon nanoparticles (Si-NPs). The Si-NPs of an average diameter of 7.42 nm was developed using an economical method from locally available sand. The impregnated Si-NPs onto the Ni-MOF nanosheets using a green approach affording bandgap engineering. The results indicate that highly pure Si-NPs was successfully developed in scalable quantity from locally available sand. Various mass loadings in the range of 1–7.5 wt% of developed Si-NPs were decorated on the surface of Ni-MOF nanosheets yielding new nanophotocatalysts recording bandgap of 2.68 eV and achieving reduction by ~ 50% compared to Ni-MOF sheets free Si-NPs. The attained photocatalysts were then exploited for photocatalytic degradation of various organic pollutants, including Malachite Green (MG), Crystal violet dye (CV), and Tetracycline (TC) and demonstrated excellent degradation efficacy. The photocatalytic degradation of MG, CV, and TC, achieving degradation efficiencies of 91.7%, 86.8%, and 95.2%, respectively, representing a significant enhancement compared to silicon-free Ni-MOF (MG: 45.8%, CV: 39.6%, TC: 44.6%). Photocatalytic degradation factors such as initial organic pollutant concentration, pH, catalyst dose, reaction time, and temperatures were also studied. Importantly, the adsorption isotherms, kinetics, and thermodynamic parameters were also investigated. The biological phytotoxicity study shows no significant differences of plant height between the plants irrigated with regular water (19.90 ± 1.7 cm) and those irrigated with water treated with the developed photocatalyst (18.86 ± 2.92 cm) in comparison to polluted water irrigate plant (14.49 ± 2.10 cm). Additionally, the antimicrobial study confirmed the inhibitory actions against bacterial strains, achieving antibacterial inhibition zone of 47.7 mm and 30 mm, against *S. aureus* and *E. coli*, respectively. Interestingly, the reusability and economic feasibility indicates that the developed catalyst can be reused efficiently for up to 5 cycles without significant decrease in photocatalyst efficiency and cost of photocatalyst, which is sufficient for treating approximately 20 m^3^ of wastewater efficiently, is about $154.50, respectively.

**Supplementary Information:**

The online version contains supplementary material available at 10.1038/s41598-025-17952-9.

## Introduction

The past few decades have seen a dramatic increase in the world’s water crisis as a result of population growth, which has raised human activity in homes, industries, hospitals, and agriculture. As a result, a substantial volume of wastewater is produced, leading to increasing environmental pollution and lacking access to clean and safe water in some countries [1]. According to UNICEF reports, safe water is inaccessible to hundreds of millions of people, especially in low-income African countries. As a result, there is a growing demand for clean water production technologies, such as water desalination and wastewater treatment, in countries that are suffering from water shortages [2]. The color wastewater represents a significant portion of the wastewater problem. The primary contributors to water coloration are toxic dyes and antibiotics released from various industrial activities. Every year, approximately 177,000 tons of dyes and 54,000 tons of antibiotics are indiscriminately discharged into global water resources [3, 4]. These materials pose a serious danger to humans, animals, plants, and the entire ecosystem. Their danger stems from their stable structures, which is hardly decompose in the environment due to the presence of auxochromes and chromophores alongside aromatic rings [5]. Accordingly, there are several techniques have been reported for wastewater treatment, including adsorption [6, 7], advanced oxidation process [1, 8–12], separation by membranes [13, 14] and separation by nano-filters [15, 16]. Photocatalysis is a promising advanced oxidation process that involves the conversion of photonic energy into chemical energy. The source of photonic energy can be obtained from ultraviolet light, sunlight, or visible light. The photocatalytic reaction aims to produce highly active species, such as holes (h^+^) and electron (e^−^) pairs, using photon energy that is equal to or greater than the bandgap (E_g_) energy [8]. Theoretically, the reaction initiated when photons from the light source hit the electrons in the valence band (VB), causing an excitation and transferring the excited electrons into the conduction band (CB), thereby leaving holes (h^+^) in the VB [8]. The electrons and holes both move to the surface of the photocatalyst, where the holes form hydroxyl radicals (^·^OH) by oxidizing water (H_2_O) [8]. These active species initiate a chain reaction to oxidize organic pollutants [8]. On the other hand, superoxides (^·^O_2_^−^) form when the electrons are donated to O_2_, which is reduced to a lower valence state and deposited on the photocatalyst surface [8]. This oxidation/reduction (Redox) process effectively degrades organic pollutants, converting them into simpler and more eco-friendly compounds [8, 17]. The successful and highly performance photocatalyst needs a narrow bandgap and a high surface area structure to achieve optimal results [17]. On the other hand, metal–organic frameworks (MOFs) are high surface area hybrid materials made up of organic linkers that connect metal clusters [18]; the wide surface area accepts MOFs with a wide range of chemical functionalities and applications. MOFs are promising materials in the adsorption and separation of gases [18], sensing [19, 20], microelectronics [21], optics [22], ion conductivity [23], pollutant sequestration, contrast agents [21], drug delivery [24], micromotors [25], bioreactors [26], and advanced photocatalytic applications [27, 28]. Interestingly, 2D Ni-MOF nanosheets are special class of MOFs materials with unique structural properties, surface areas, tunable functionalities, relatively low bandgap, and strong stability [29]. This is in addition to various synthesis routes such as sonochemical [30], microwave [31] and solvothermal process [32]. Recently, 2D Ni-MOFs was used for removal of organic dyes via adsorption of organic pollutants [33–35]. However, a few studies have exploited 2D Ni-MOF for photodegradation of organic pollutants upon narrowing down the bandgap between the HOMO and LUMO orbitals through various approaches which generally denoted as bandgap engineering process [36]. Recently, flowerlike Bi_2_WO_6_ was used in conjunction with 2D Ni-MOF nanosheets (80:20 wt%), achieving bandgap enhancement ranging from 2.23 to 2.67 eV and in turn improve degradation [37]. Interestingly, silicon is interesting semiconductor and extensively used in advanced technology, including the manufacturing of electronic chips and solar cells, due to its abundant availability source from commercial sand, cost-effectiveness, and unique bandgap properties (1.12 eV) [38]. Therefore, silicon (Si) is considered as an excellent choice for bandgap engineering applications [39]. Thus, rational approach for green fabrication of cost-effective and efficient nanophotocatalyst with narrow bandgap energy is highly required for efficient purification of wastewater based organic pollutants. Hence, novel, scalable, and green nanophotocatalyst with an engineered narrow bandgap based on spherical Si nanoparticles derived from sand was developed. The developed nanophotocatalyst achieved superior photocatalytic degradation (~ 95% degradation efficiency with operating conditions of 0.25 g/L catalyst dose, 50 ppm pollutant concentration, 180 min reaction time, room temperature and pH 4.1 under visible light) compared to reported systems [37, 40–45]. Thus, a novel photocatalyst based on Ni-MOF and Si-NPs using a green impregnation method was successfully fabricated. Various concentrations of Si-NPs were employed to investigate the optimal ratio between Ni-MOF and Si-NPs. The crystal, morphological, physicochemical, and optical properties of the produced catalysts were characterized. The prepared catalysts were utilized for the photocatalytic degradation of Malachite Green (MG), Crystal violet (CV), and Tetracycline (TC) pollutants under visible light. Additionally, the different parameters affecting the MG degradation rate, including contact time, MG concentration, catalyst dose, pH, and temperature, were studied and optimized, and a photocatalytic degradation mechanism was proposed.

## Materials and methods

### Materials

Nickel (II) nitrate hexahydrate (Ni (NO_3_)_2_·6H_2_O) and terephthalic acid (1,4-benzenedicarboxylic acid, H_2_BDC) were obtained from Merck (Germany) and handled as received. Malachite Green (MG, C_23_H_25_N_2_·Cl, λ_max_ = 619 nm) and Crystal violet Dye (CV, C_25_N_3_H_30_Cl, λ_max_ = 595 nm) were supplied by Fluka (Switzerland). Tetracycline (TC, C_22_H_24_N_2_O_8_, λ_max_ = 362 nm) was provided by Sigma Aldrich Company Ltd (USA). N,N-Dimethylformamide (DMF), anhydrous ethanol (EtOH), methanol (MeOH), magnesium powder (Mg powder), hydrochloric acid (HCl), and sodium chloride (NaCl) were of analytical grade and purchased from various suppliers. Additionally, the sand used in the study was sourced from Egyptian sand, which is widely available.

### Synthesis of Si nanoparticles (Si-NPs)

The Si-NPs were prepared according to modification of the reported methods [46, 47]. The synthesis details; the sand was collected, cleaned, and then ground in a ball mill to reduce its particle size. Then, a mixture of ground sand (5 g) and magnesium powder (4 g) and NaCl (5 g) in a mass ratio of 1:0.8:1 for sand, Mg, and NaCl, respectively, was heated in an oven at 750 °C for 5 h. Afterwards, the attained product composed of silicon and magnesium oxide (MgO) was washed with hydrochloric acid (1M HCl) several times and then NaOH solution and followed by DI water . Afterward, dried in an oven yielding Si-NPs as presented in Scheme S1.

### Synthesis of 2D Ni-MOF sheets

2D Ni-MOF nanosheets was synthesized based on modification of the reported procedure [32]. Briefly, 2.2 g of Ni (NO_3_)_2_·6H_2_O and 1.98 g of H_2_BDC were dissolved in a mixed solvent containing 70 mL of DMF, 5 mL of EtOH, and 5 mL of H_2_O. This mixture was sonicated at room temperature and stirred until the solution became transparent. The resulting solution was then transferred in to a PTFE-lined stainless-steel vessel (100 mL) and heated to 120 °C for 12 h. Afterwards, the green precipitate was allowed to cool to room temperature, collected by centrifugation, washed three times with DMF followed by anhydrous EtOH, and then dried in an oven at 80 °C for 12 h.

### Synthesis of Si-NP-Ni-MOF nanophotocatalyst

**Scheme 1:**
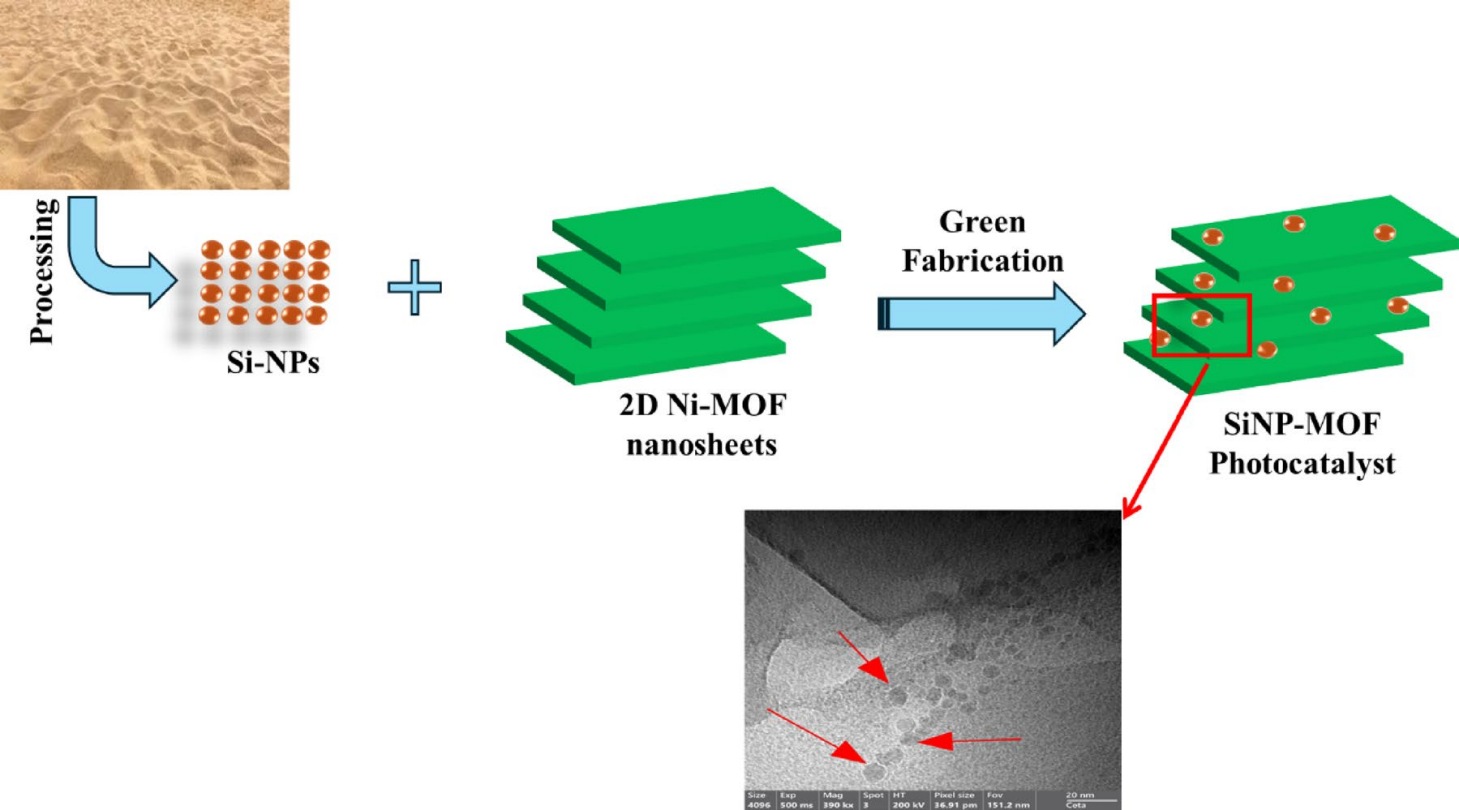
Schematic diagram illustrating the green impregnation of Si-NPs onto 2D Ni-MOF sheets.

In two glass beakers each one contains 50 mL DI water disperse different mass ratios of Si-NPs and 2D Ni-MOF nanosheets individually (Table 1) and then they sonicated individually for 10 min. Afterwards, two dispersions are then combined and sonicated for an additional 10 min and stirred for another 2 h to ensure that the Si-NPs is homogeneously dispersed over the MOF surface (Scheme 1). Five different photocatalyst nanocomposites were developed as indicated in Table 1. The developed nanophotocatalyst were denoted as SiNP-MOFx, as SiNP refers to Si-NPs, MOF refers to 2D Ni MOF nanosheets and x refers to the mass ratio of Si-NPs.

**Table 1:** Illustrates the various compositions of developed photocatalyst.

Photocatalyst Code	Mass % of Ni-MOF	Mass % of Si-NPs
SiNP-MOF0	100	0
SiNP-MOF1	99	1
SiNP-MOF2.5	97.5	2.5
SiNP-MOF5	95	5
SiNP-MOF7.5	92.5	7.5

### Photocatalytic degradation experiment

The photocatalytic degradation process of different pollutants was carried out under an atypical LED lamp 100W. Typically, 5 mg of developed photocatalyst was dispersed individually in 20 mL of MG (50 ppm), TC (10 ppm), and CV (10 ppm) in a 50 ml beaker and sonicated for 30 s, then stirred in the dark for 30 min at room temperature. Then, about 3 mL of solution were collected and centrifuged at (15,000 rpm) and measured using a UV–VIS spectrophotometer at λmax = 619, 595, and 362 nm for MG, CV, and TC, respectively. Then, the collected sample was returned to the beaker after redispersion of the nanophotocatalyst and the photocatalytic degradation was started under the LED lamp at a distance of 20 cm between the lamp and the solution surface. The degradation temperature was kept at 25 ± 2 °C using a specific unit that was specially designed and fabricated locally by GANNTEC Company for this purpose consisting of multi stirrer (15*1) caped with water heat exchanger unit and temperature regulator unit (Fig. S1). After 180 min of irradiation, a 3 mL aliquot of the reaction mixture was collected, centrifuged at 15,000 rpm, and analyzed by UV–Vis spectrophotometry to determine the residual pollutant concentration. The photocatalytic degradation percentage was calculated using Eq. 1.

1 $$Photocatalylic\,degradation \left( \% \right) = \frac{{C_{o - } C_{t} }}{{C_{o} }} \times 100$$

where the C_0_ and C_t_ are the concentration of a pollutant at time = 0 and after time = t, respectively [44].

### Characterization

The crystal structure for the prepared materials was detected using X-ray diffraction (XRD) from PAN analytical equipment Co. model X’Pert PRO with a Secondary Monochromator and Cu radiation (λ = 1.542Å) at accelerating voltage 45 kV, current, 35 mA, and scanning speed 0.04°/sec in range of 2θ from 2 to 80°. The Fourier Transform Infrared (FT-IR) analysis was carried out using FTIR Model IRPrestige-21 produced by Shimadzu Company, in spectral range 4000-400cm^−1^. The morphological structure was characterized by using scanning electron microscopy (SEM) attached to an Energy Dispersive X-ray Unit (EDX) model Quanta 250 FEG (Field Emission Gun) made by FEI Company. The High-resolution Transmission Electron Microscope (HR-TEM) imaging was done using the Thermo Scientific model Talos™ F200i TEM at an accelerating voltage of 200 kV. The specific surface area (SSA) using Bruner-Emmett-Teller method (BET) and pore volume of the synthesized materials were measured using BELSORP-mini-X by Microtrac BEL Co. The elemental composition and chemical state of materials were investigated using X-ray photoelectron spectroscopy (XPS) analysis via ESCALAB 250Xi by Thermo Scientific Co. model ESCALAB 250Xi XPS. The photocatalytic degradation activity for the prepared catalysts was detected using Jenway 6705 UV–Vis Spectrophotometer ranging from 200 to 1000 nm.

## Results and discussion

### Crystal, structural and morphological characterization of developed nanophotocatalysts

**Fig. 1:**
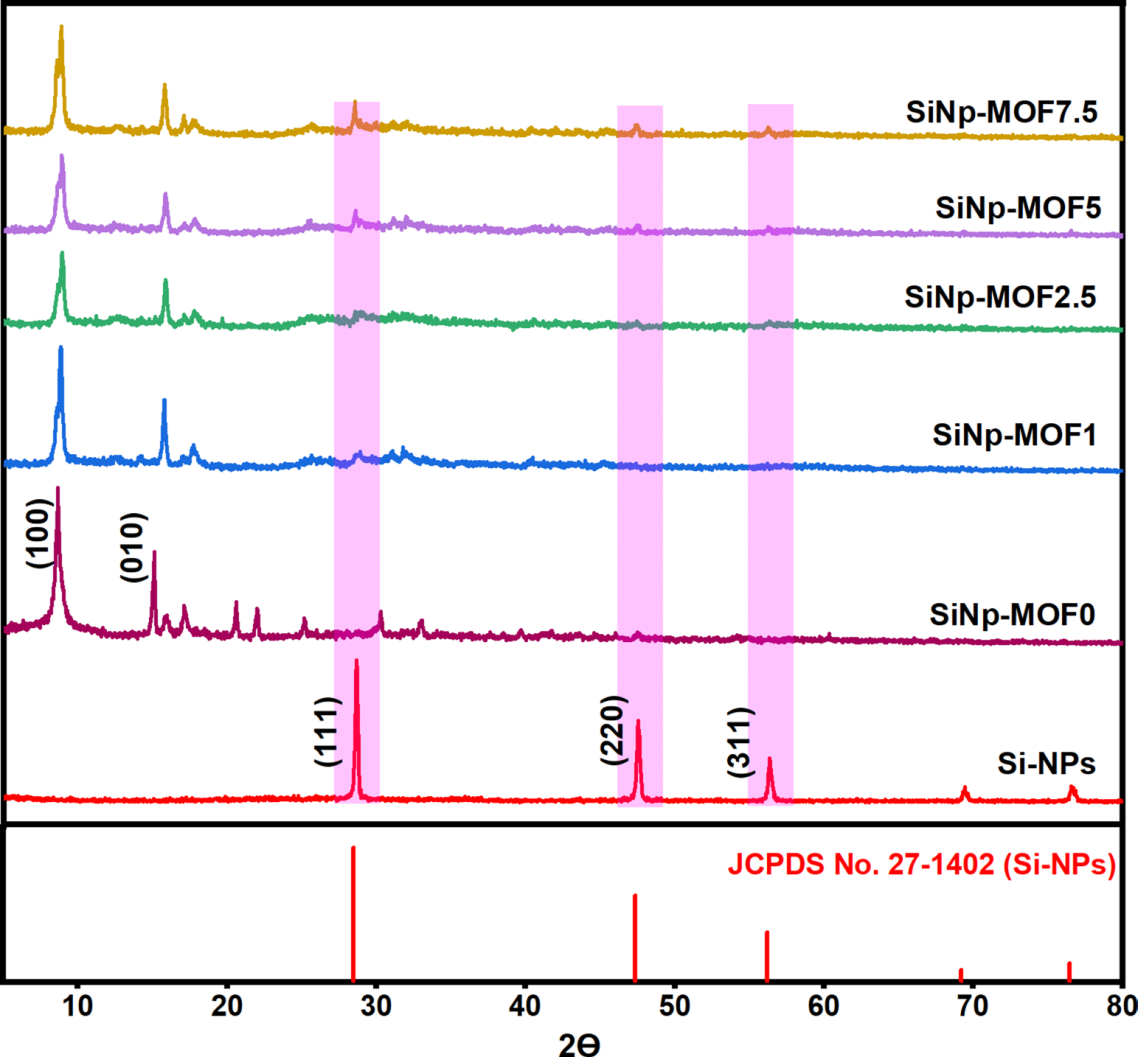
XRD patterns of the prepared Si-NPs and different SiNP-MOF nanophotocatalysts concentrations.

Green approach was developed for efficient fabrication of nanophotocatalysts composed from Si-NPs derived from commercial sand and then, uniformly dispersed on 2D Ni MOF nanosheets surface as depicted in Scheme 1. The newly developed nanocomposites record narrow bandgap compared to 2D Ni MOF nanosheets and thus achieved efficient photocatalytic degradation for various organic pollutants affording scalable approach for purifying aqueous wastewater. The developed nanophotocatalysts were prepared in various compositions to tailor the influence of Si-NPs in bandgap engineering process. Thus, various mass loadings of Si-NPs of 1, 2.5, 5 and 7.5 wt% were used for decoration of 2D Ni MOF nanosheets with the aid of ultrasonication. The developed nanophotocatalysts and prepared, MOF and Si-NPs were elucidated using various techniques such as XRD, SEM and TEM. The XRD patterns for Si-NPs and SiNP-MOF 0, 1, 2.5, 5, and 7.5% are illustrated in Fig. 1. The XRD peaks for Si-NPs were recognized at 2 ϴ angle of 28.65°, 47.5°, 56.32°, 69.35°, and 76.54°, which related to planes (111), (220), (311), (400), and (331), respectively; these patterns can be readily indexed to a cubic phase of silicon (JCPDS No. 27-1402) [48, 49]. The same planes were corroborated in the selected area of electron diffraction pattern (SAED) (Fig. S2) and consistent with the previous report and indicates the Si-NPs have a cubic face-centered lattice structure [50]. This affirms the successful synthesis of Si-NPs. On the other hand, the characteristic diffraction peaks for 2D Ni-MOF (SiNP-MOF0) were noticed at 2 ϴ angle of 8.6, 13.8, 15.8, 17.1, and 21.9◦, which correspond to (100), (010), (101), (210), and (112) crystal planes, respectively (Fig. 1) [32, 51]. The observed XRD pattern found a good match with CCDC No. 638866 [51, 52]. For the developed nanophotocatalysts, the XRD pattern for SiNP-MOF1 and SiNP-MOF2.5 displayed no change in the crystal structure compared to SiNP-MOF0 (2D Ni MOF nanosheets), and this could be due to the low concentration of Si-NPs decorated on MOF surface, which was under the detection limit of XRD. Interestingly, upon inclusion of 5 and 7.5 wt% of Si-NPs in the samples of SiNP-MOF5 and SiNP-MOF7.5, the XRD pattern illustrates detectable clear peaks at 28.65°, 47.5°, and 56.32° which are characteristic to Si-NPs and confirms the success of fabrication process of SiNP-MOF. Additionally, no detectable change was noticed in the crystal structure of MOF, indicating that the impregnation of Si-NPs occurred on the surface of the sheets without altering the crystal structure of the Ni-MOF.

**Fig. 2:**
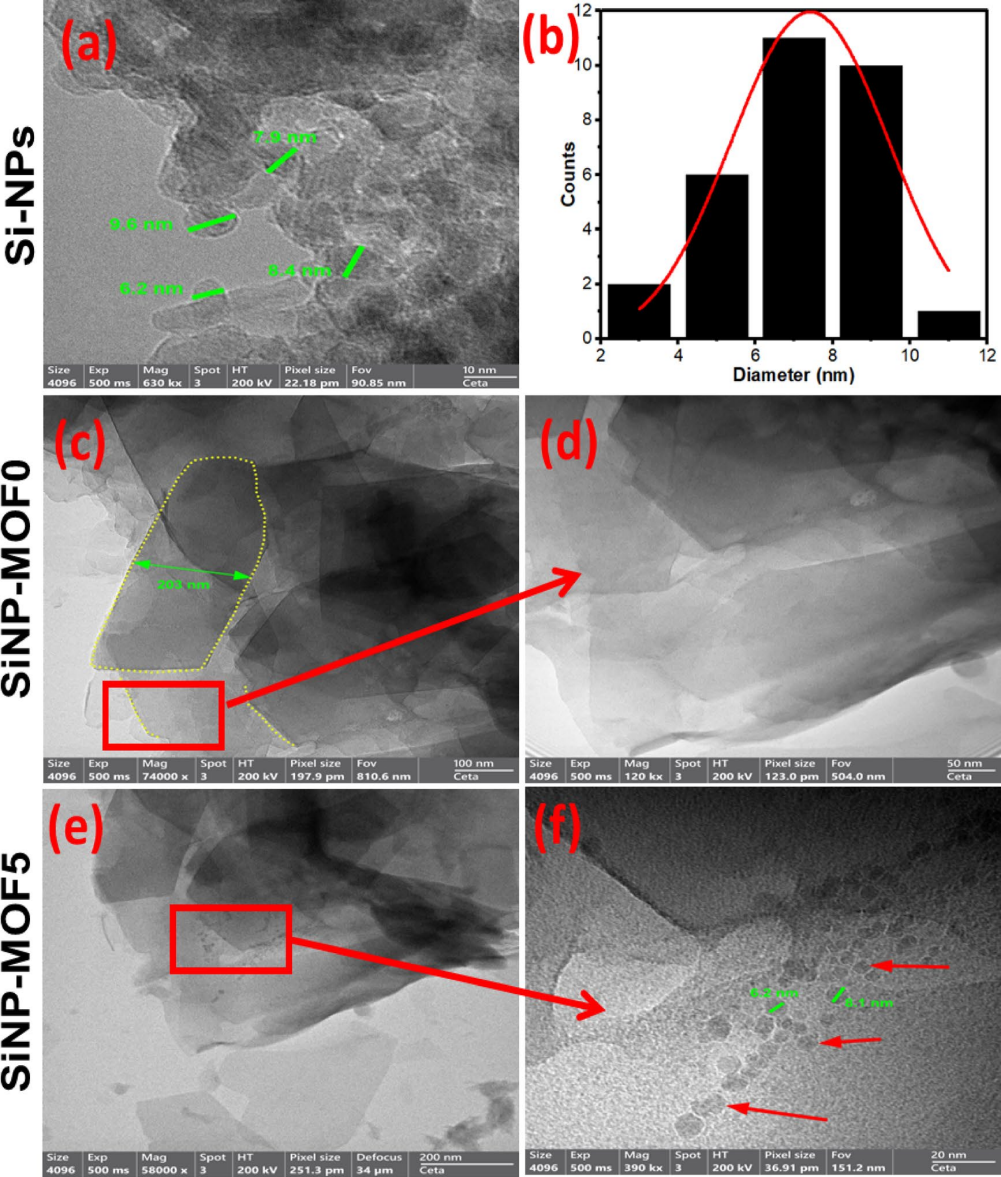
HR-TEM images of Si-NPs (**a**), particle size histogram distribution of Si-NPs (**b**), SiNP-MOF0 (**c**, **d**), and SiNP-MOF5 (**e**, **f**) samples.

On the other hand, the surface morphology and nanoscale size of Si-NPs, 2D Ni MOF nanosheets and their corresponding nanocomposites were studied using SEM and TEM. Fig. S3a shows SEM image of Si-NPs which illustrates porous structure in nanoscale dimension was attained, the porous structures on the surface of the produced Si-NPs was stemmed from adding NaCl during the magnesiothermic reduction process [46, 53]. On the other hand, the HR-TEM of Si-NPs image confirms the nano structure and individualization of Si-NPs that appeared in a grey shade with an average particle size of 7.42 ± 0.40 nm with narrow size distribution (Fig. 2a and b). Also, the interlayer (interplanar) distance was measured using Image j software (Fig. S4), displayed d = 0.324 nm, matched with the *d* spacing of the (111) plane that presented in the XRD pattern. For 2D Ni MOF nanosheets, the HR-SEM images at magnification 100,000 X of Ni-MOF displaying a uniform structure of 2D nanosheets in 2D dimensions with a diameter ranging from 200 to 612 nm (Fig. S3b). Additionally, unevenly color-stratified layer structure in TEM images Fig. 2c and d, suggesting the layer stacking [54], which consistent with the SEM results. The thin nanosheets that appeared in the high-magnified SEM and TEM image and their expansion in 2D dimensions affords suitable surface area which are desirable for pollutant adsorption and in turn facilitates efficient photocatalytic degradation process. The surface morphology of developed SiNP-MOF5 photocatalyst was visualized in Fig. S3c which displayed 2D Ni-MOF nanosheets decorated with spherical Si-NPs in uniform dispersion form. The high magnification TEM image clearly visualized the uniform dispersion of individual spherical Si-NPs of an average size of 7.42 nm over 2D Ni MOF nanosheets (Fig. 2e and f). Thus, affirms the successful fabrication of nanophotocatalyst and an efficient decoration process of Si-NPs in uniform regime over 2D Ni MOF nanosheets was carried out.

**Fig. 3:**
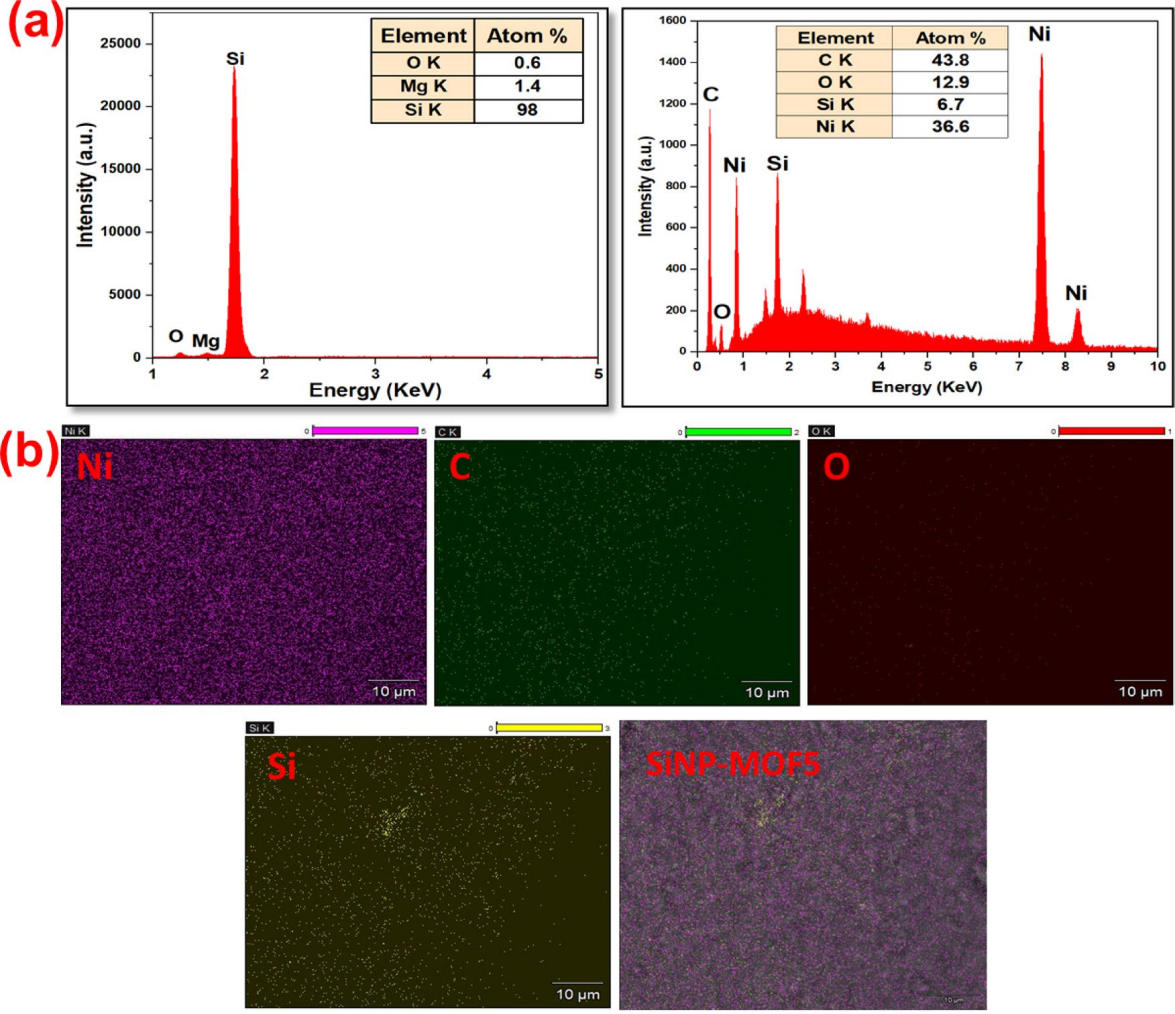
EDX (**a**) and Map EDX (**b**) of SiNP-MOF0 and SiNP-MOF5 samples.

The developed nanophotocatalyst composition and dispersion of Si-NPs over 2D Ni-MOFs surface was further investigated using EDX as illustrated in Fig. 3. The elemental composition of developed Si-NPs records 98 At. % in conjunction with traces of oxygen, magnesium of 0.6 and 1.4 At%, respectively, (Fig. 3a) affirming high purity of attained Si-NPs. However, the EDX for SiNP-MOF5 reflecting the existence of carbon, oxygen, nickel and silicon of 43.8, 12.9, 36.6 and 6.7At%, respectively. The mapping mode was also carried for SiNP-MOF5 to elucidate the dispersion of elements in the nanocomposite. The results displayed uniform dispersion of Si-NPs over MOF nanosheets surface (Fig. 3b).

### Textural and structural properties of Si-NPs, MOF nanosheets and SiNP-MOF5

The porous structure of the prepared materials was also studied using nitrogen adsorption–desorption process. Fig. S5 illustrates the N_2_ adsorption–desorption isotherm of Si-NPs, SiNP-MOF0, and SiNP-MOF5 samples. The three samples revealed a type IV isotherm with hysteresis loop, which are characteristic to mesoporous structure of the samples [55]. The BET specific surface area (SSA) for Si-NPs was found to be 31.02 m^2^g^−1^ with a total pore volume (TPV) of 0.2 cm^3^ g^−1^ that confirmed the mesoporous structure of Si-NPs. However, the specific surface area of SiNP-MOF0 was found to be 17.2 m^2^g^−1^ (Fig. S5) with TPV of 0.23 cm^3^ g^−1^[56]. The surface area of SiNP-MOF5 decreased to 5.8 m^2^g^−1^, as indicated in Table S1, which can be related to the blocking of MOF pores by Si-NPs that were impregnated on its sheets (Fig. S5).

**Fig. 4:**
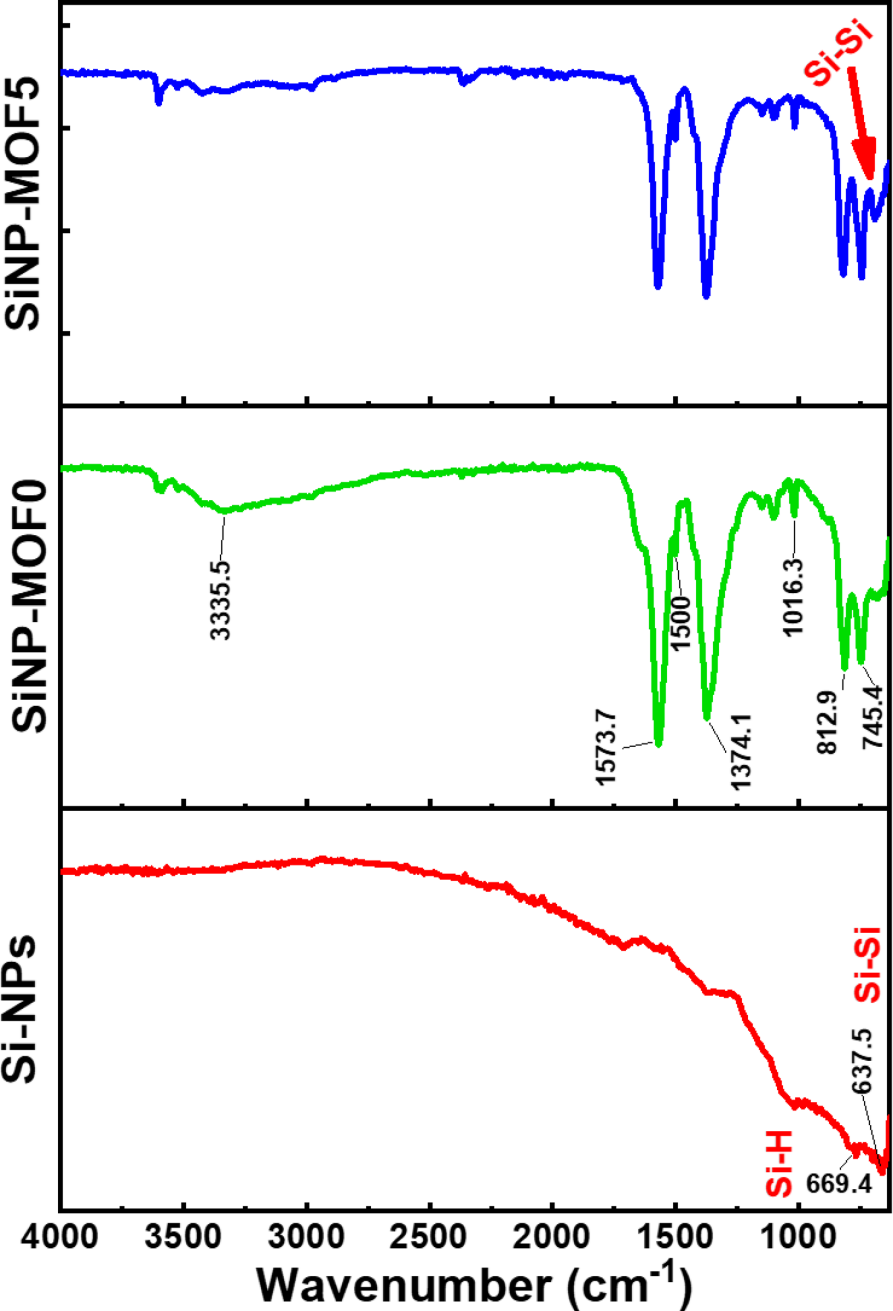
FTIR spectra of Si-NPs, SiNP-MOF0, and SiNP-MOF5 samples.

The FT-IR spectrum of samples Si-NPs, SiNP-MOF0, and SiNP-MOF5 are shown in Fig. 4. The FTIR spectra for Si-NPs shows no sign for the Si–O–Si bond, which is usually located at 1070 cm^−1^ [57, 58], thus, affirms the full reduction of SiO_2_. Moreover, the small band noticed around 637 cm^−1^ is assigned to the stretching vibration mode of the bulk Si–Si bond [59]. Additionally, the small band observed at 669 cm^−1^, corresponds to the Si–H vibration band which stemmed from the HCl used in leaching process [59–61]. For SiNP-MOF0, the FTIR spectrum display a prominent peak at 3355 cm⁻^1^, indicative of O–H stretching vibrations from surface-adsorbed water molecules [32, 51], furthermore the characteristic absorption bands of 2D Ni MOF were observed at 1573 and 1374 cm^−1^, are assigned to antisymmetric (–COO^−^) and symmetric (–COO^−^), respectively [62, 63]. The peak at 1500 cm⁻^1^ arises from para-aromatic CH group (Fig. 4) [32, 51]. The absorption band located at ~ 1000 cm^−1^ is related to the stretching vibrations of metal–oxygen [60]. The absorption band, positioned at 754 and 812.9 cm^−1^ are characteristic to the para di substitution of the benzene ring[61]. Moreover, for SiNP-MOF5 the noticed small new band around at 640 cm^−1^ is corresponding to the Si impregnation into the Ni MOF sheets which is assigned to the Si–Si bond that was shown in the Si-NPs spectrum (Fig. 4).

### Optical properties

**Fig. 5:**
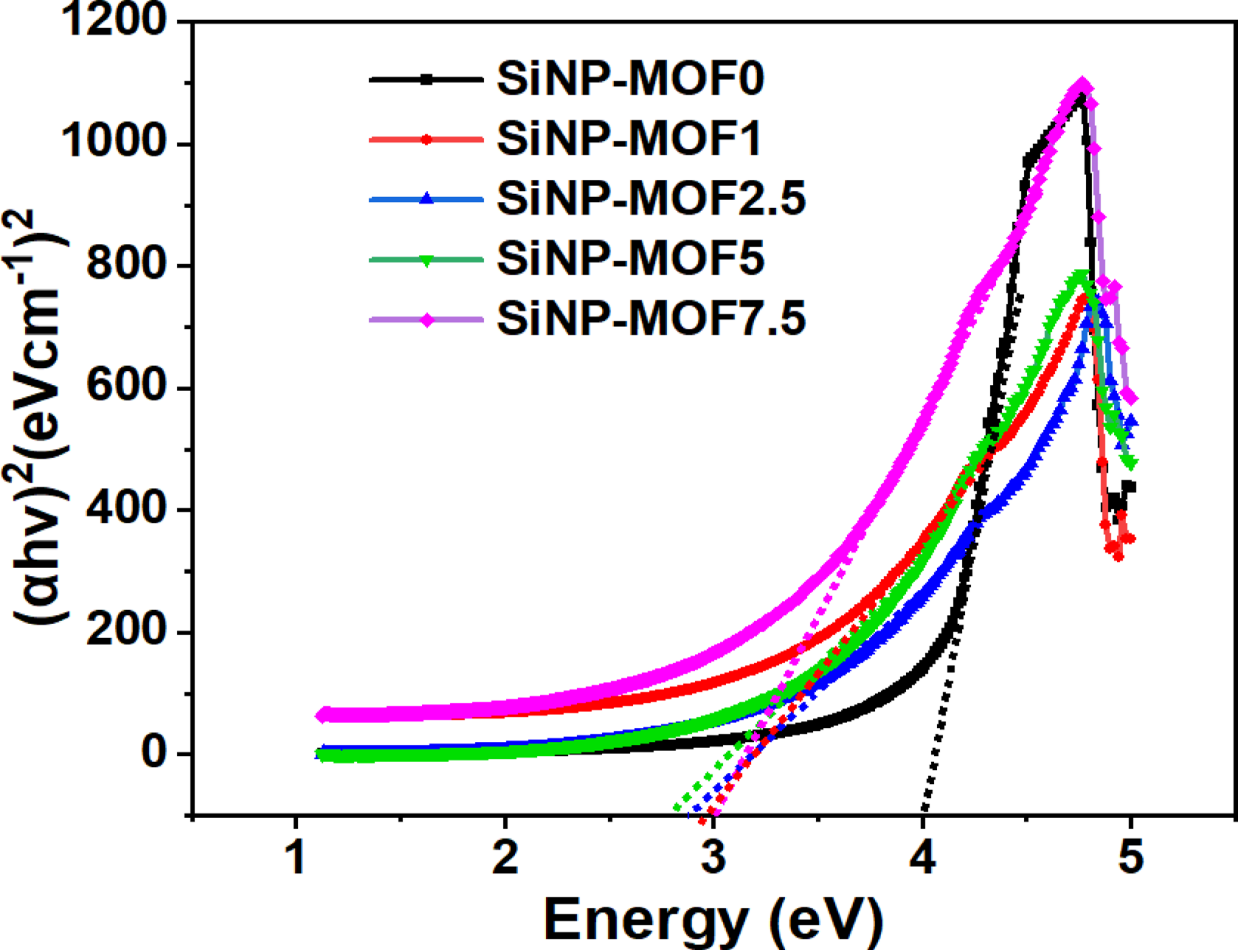
Direct band gap energy (Eg) from the Tauc plot for SiNP-MOF0, 1, 2.5, 5, and 7.5 samples.

The study of band gap energy (E_g_) plays a key role in the photocatalytic degradation process because it can precisely predict the photocatalyst’s performance. The bandgap energy for samples SiNP-MOF 0, 1, 2.5, 5, and 7.5 samples were determined using the Tauc plot (Fig. 5), following the established methods reported in the previous literatures [64–68], the band gap energy (E_g_) of samples can be calculated via Eq. 2 [69]:

2 $$\left( {\alpha hv} \right)^{2} = K \left( {{\text{h}}v - { }E_{g} } \right)$$

where α is refers to the absorption coefficient being a function of wavelength, K is the proportionality constant, h is the Planck constant, ⱱ is frequency, and E_g_ is bandgap energy. As shown in Fig. 5, the calculated band gap for 2D Ni-MOF nanosheets (sample SiNP-MOF0) was found to be around 3.97 eV which agrees with that reported literature [40]. However, with the incorporation of the Si-NPs on the surface of Ni-MOF nanosheets, bridging phenomena has been occurred and new empirical bandgap has been created achieving new bandgaps of 2.9, 2.81, 2.68, and 3 eV for SiNP-MOF1, SiNP-MOF2.5, SiNP-MOF5 and SiNP-MOF7.5, respectively. This tailoring in bandgap was in response to mass loadings of anchored Si-NPs, which could afford superior photocatalytic degradation efficiency of different organic pollutants under visible light especially in sample SiNP-MOF5 that achieved the lowest bandgap.

### X-ray photoelectron spectroscopy (XPS)

**Fig. 6:**
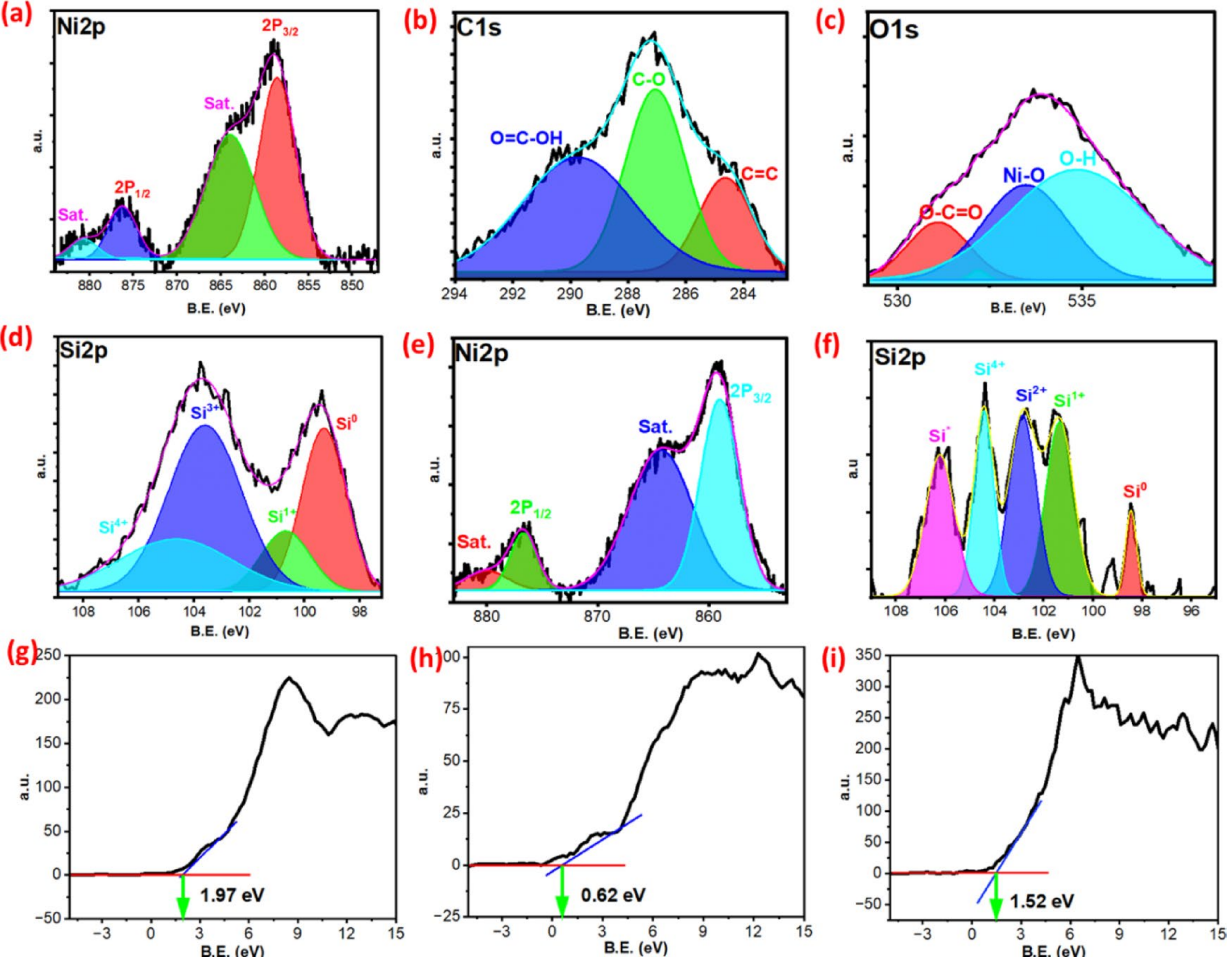
Individual high-resolution XPS spectra of Ni2p (**a**), C1s (**b**), and O1s (**c**) for SiNP-MOF0. XPS for Si2p (**d**) in Si-NPs, Ni2p (**e**), Si2p (**f**) for SiNP-MOF5. XPS Valence band position for SiNP-MOF0 (**g**), Si-NPs (**h**), and SiNP-MOF5 (**i**).

The XPS analysis was used to further study the composition and chemical states of prepared SiNP-MOF0, Si-NPs, and the best photocatalytic performance sample SiNP-MOF5. Fig. S6a shows the survey scan spectrum SiNP-MOF0, which confirms the Ni, C, and O elements signals. However, Fig. 6a shows the XPS spectrum of Ni 2p; two peaks at binding energy (BE) of 875.40 and 856.10 eV correspond to Ni 2p1/2 and 2p3/2, respectively, which indicate the oxidation state of Ni are + 2 [32], and two peaks at about BE = 881.20 and 863.20 eV are shake-up satellite peaks [70]. On the other hand, the XPS for C 1s displayed in Fig. 6b shows a wide peak fitted to 284.8, 287.6, and 289.7 eV, which represent the C=C, C–O, and carboxylate carbon (O=C–OH) bonds, respectively, which confirm the chemical structure of H_2_BDC used in 2D Ni-MOF preparation [70, 71]. The O 1s spectra Fig. 6c shows three fitted peaks at 535.9, 534.3, and 532.7 eV bonds that related to O–H, metal–oxygen bonds (Ni–O), and O–C=O functional group [72, 73]. Figure 6d records the XPS spectra of Si 2p of the prepared Si-NPs; the strong peak located at 99.11, was assigned to Si^0^, which confirms the reduction of SiO_2_ during the magnesiothermic reduction process [74]. Another fitting peaks at BE = 100.4, 103.2, 104.3 eV are related to the different oxidation state of silicon Si^1+^, Si^3+^, and Si^+4^, respectively [75], appearance of different oxidation states indicated to formation of oxide layer on the bulk silicon surface. The limitation of XPS is inability to determine the chemical composition except in the first layer only, which does not exceed 10 nm [76–78], but the bulk structure of Si^0^ was confirmed via XRD, TEM, EDX, and FTIR. The XPS of Ni2p for SiNP-MOF5 (Fig. 6e) revealed a slight shift in the Ni 2p1/2 and 2p3/2 peaks to 876.80 and 858.70 eV, respectively, which may be related to the Si-NPs. Although the loading percentage of Si-NPs is very low, the XPS survey for SiNP-MOF5 (Fig. S6b) detect the Si-NPs impregnation on the surface of 2D Ni-MOF sheets, which fitted to small four peaks represent the different oxidation states of Si (Fig. 6f). The valence band (V_B_) of SiNP-MOF0, Si-NPs, and SiNP-MOF5 is illustrated based on XPS results in Fig. 6g, h, and i, respectively. The V_B_ for SiNP-MOF0 was found to be at 1.97 eV, which is consistent with previous literature that reports the V_B_ for Ni-MOF to be around 2 eV [79–81]. Based on the bandgap studies from the Tauc plot (3.97 eV), the conduction band (CB) for SiNP-MOF0 is estimated to be at − 2 eV. Additionally, the V_B_ for Si-NPs was observed at 0.62 eV, which is close to the value reported by Wang et al. (2004), who measured the V_B_ for bulk Si using the same technique, reporting a V_B_ of 0.51 eV and a bandgap of 1.11 eV [82]. According to previous studies indicating the indirect bandgap of silicon at 1.12 eV [83–85], the CB of Si-NPs is estimated to be at − 0.5 eV. Furthermore, the VB for SiNP-MOF5 was shifted to a lower value of 1.52 eV, confirming the effect of Si-NPs in engineering the bandgap. Based on our calculations for the bandgap of SiNP-MOF5 (2.68 eV), the CB is estimated to be at  − 1.16 eV.

### Photocatalytic performance study

**Fig. 7:**
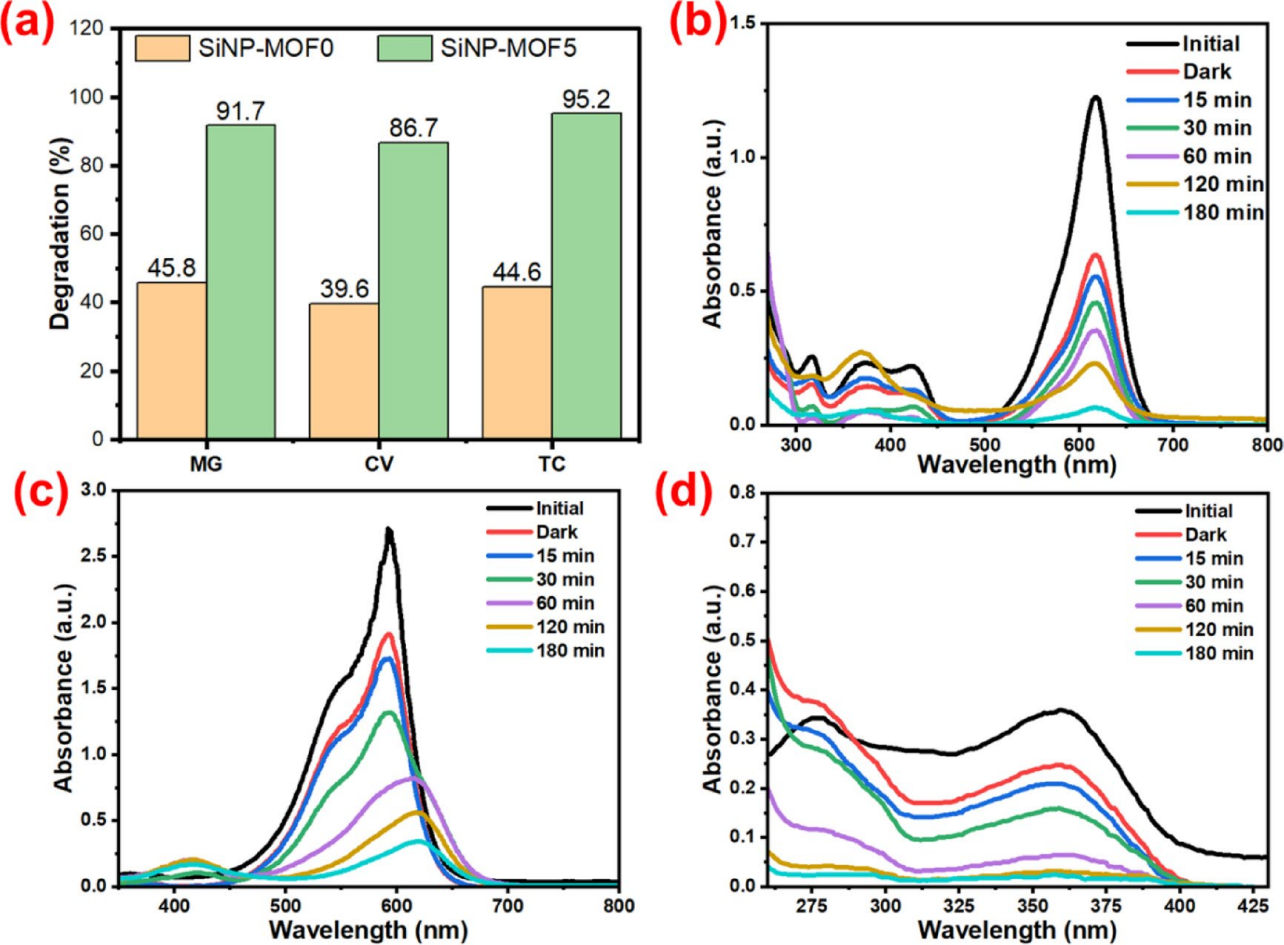
Photocatalytic degradation efficiency of SiNP-MOF0 and SiNP-MOF5 toward MG, CV, and TC pollutants (**a**), Time-dependent UV–Vis spectra of degradation of MG dye (**b**), CV dye (**c**), and TC (**d**) using SiNP-MOF5 photocatalyst.

Photocatalytic degradation performance for the developed nanophotocatalysts against various organic pollutants such as MG, and CV dyes beside TC as pharmaceutical pollutant was studied. Initially SiNP-MOF0 (Si-NPs free) and SiNP-MOF5 nanophotocatalyst were used for the study against the different pollutants. The SiNP-MOF5 was selected for general study, due to its tailored narrow bandgap and economic point of view (Fig. 7a). The operating conditions include an initial pollutant concentration of 50 ppm for MG and 10 ppm for both CV and TC with photocatalyst dosage of 0.250 g L^−1^ at pH = 4.1 (Dye pH), time = 180 min, and 25 °C under visible-light irradiation. The photocatalytic degradation efficiency of SiNP-MOF0 for MG, CV and TC was found to be 45.8%, 39.6%, and 44.6%, respectively. On the other hand, for SiNP-MOF5 nanophotocatalyst achieved superior photocatalytic degradation efficiency recording 91.7, 86.8, and 95.2% for MG, CV, and TC, respectively as illustrated in Fig. 7b-d. The huge difference in photocatalytic degradation efficiency between the SiNP-MOF0 and SiNP-MOF5 illustrates the influence of Si-NPs in narrowing down the bandgap in bandgap engineering process of 2D Ni-MOF. Thus, facilitates the potential degradation process upon the absorption of visible light. These results encourage us to further study on the several operational parameters that affect the photocatalytic degradation ability, such as contact time, photocatalyst dosage, initial pollutant concentration, pH, and temperature. Thus, MG dye was selected for further study because it had the highest concentration between the three pollutants.

#### Effect of contact time

**Fig. 8:**
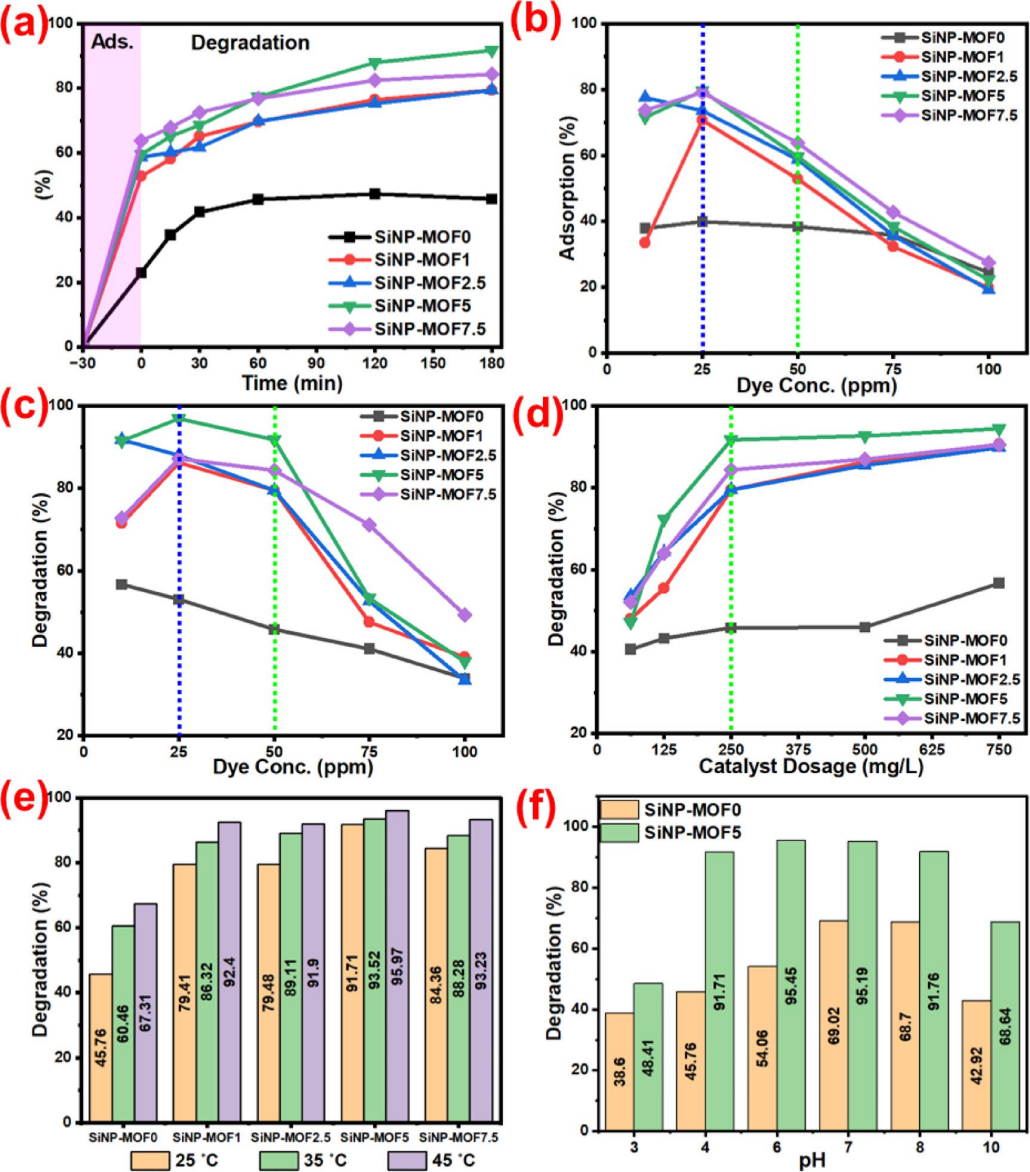
MG degradation studies: (**a**) time, (**b**) dye concentration effect on adsorption, (**c**) dye concentration effect on photodegradation, (**d**) catalyst dosage, (**e**) temperature, and (**f**) pH effects for SiNP-MOF0 and SiNP-MOF5.

The effect of contact time on MG degradation was studied at initial MG concentration = 50 ppm, pH = 4.1 (Dye pH), room temperature, and catalyst dose = 0.250 g/L, and the catalysts were first stirred with dye solution for 30 min to achieve the adsorption of MG on the catalyst surface, the results are plotted in Fig. 8a. After dark adsorption of MG, all samples adsorb a large amount of dye that could be attributed to the strong electrostatic interaction between the positively charged dye MG^+^ and aromatic moieties of Ni-MOF [86], also, the 2D structure of Ni-MOF provide surface area which facilitates the diffusion of the MG molecules between layers. Additionally, the loading of Si-NPs, which have a high surface area and mesoporous structure, will help in increasing the percentage of dye adsorbed on the catalyst surface, which attributes to the highest adsorption found in impregnated samples SiNP-MOF 1, 2.5, 5, and 7.5 recording adsorption percentage of 52.9%, 58.7%, 59.5%, and 63.8%, respectively, compared to 23% for SiNP-MOF0 which lacks of Si-NPs (Fig. 8a). Afterwards, when the degradation stage started, each catalyst had a large amount of dye adsorbed on its surface, and that facilitated the photocatalytic degradation according to the bandgap of each catalyst. The SiNP-MOF0 shows the lowest percentage of degradation (45.76%) due to its large bandgap; however, the Si-NPs impregnated samples show a higher degradation rate than SiNP-MOF0, and the percentage of photocatalytic degradation after 180 min was found to be 79.4, 79.48, 91.71, 84.36% for SiNP-MOF1, 2.5, 5, and 7.5, respectively. The slight decreasing of photocatalytic degradation in SiNP-MOF7.5 could be attributed to the darkness of color that attained by increasing the Si-NPs mass which might inhibit light photons from reaching the catalyst surface, in addition to the relative increase of bandgap value for the SiNP-MOF7.5 (3 eV) compared to the SiNP-MOF5 (2.68 eV) sample.

Figure 7b illustrates the UV–Vis spectra of MG dye degradation via the highest degradation rate of SiNP-MOF5. The intensity of the characteristic peak of MG dye (λmax = 619 nm) decreased from 1.22 initially to 0.637 after adsorption, then gradually reduced by increasing the photocatalysis period, reaching the lowest intensity of 0.114, after 180 min. Similarly, the characteristic peak for CV dye at 595 nm decreased from 2.697 to 1.826 after adsorption and then dramatically dropped to 0.340 after photodegradation over the same time period (Fig. 7c). Additionally, the intensity of the characteristic peak for TC at 362 nm decreased from 0.355 to 0.276 after adsorption. Following photodegradation, the intensity further decreased to 0.016 within 180 min (Fig. 7d). Thus, affirming the 180 min reaction is the optimum time for reaching maximum photocatalytic degradation process for developed photocatalyst.

#### Effect of initial MG concentration

The effect of initial dye concentration was studied at contact time = 30 min for adsorption and 180 min for photodegradation, pH = 4.2 (Dye pH), 25°C , and catalyst dose = 0.250 g/L. Importantly to note that, the developed nanophotocatalyst affords higher adsorption capacity besides its photocatalytic activity. Hence, the adsorption percentage during the first 30 min in the dark stage beside the main study on the photodegradation percentage for 180 min was conducted. Figure 8b describes the effect of initial dye concentration on the adsorption stage during the 30 min adsorption in dark period and the results displayed that adsorption increases by increases of the dye concentration from 10 to 25 ppm and the adsorption efficiency at 25 ppm was found to be 39.94, 70.61, 73.53, 79.61, and 79.10% for samples SiNP-MOF0, 1, 2.5, 5, and 7.5, respectively. This could be due to the adsorption sites’ unsaturation in the photocatalyst surface which encourage the rapid adsorption of dye at low concentrations. However, upon increasing the dye concentration, the ability of catalysts to adsorb further amount of dye molecules is dramatically decreased because of the occupation of the adsorption sites and the repulsion force between the positive charge of dye molecules on the adsorption sites and new dye molecules dispersed in solution [6, 7] as displayed in Fig. 8b. Figure 8c illustrates the effect of initial MG concentration on the photocatalytic degradation percentage for 180 min, the results displayed that the highest photocatalytic degradation was achieved with initial MG concentration of 25 achieving degradation of 52.99, 86.20, 87.98, 96.85, and 87.22% for SiNP-MOF0, 1, 2.5, 5, and 7.5, respectively. These values were slightly changed via increasing the MG concentration to 50 ppm, however, any further increase of MG concentration cause a significant drop in the degradation efficiency. This phenomenon could be stemmed from the fact that, the high concentration of MG hinders the penetration of light photons to the active sites on the catalyst surface [44], thus reducing the total active radicals generated for MG photodegradation. Additionally, as MG concentration increased, more organic intermediates and end products were produced. These products competed with the original MG molecules for adsorption sites on the active sites of the photocatalyst, lowering the efficiency of photocatalytic degradation [87]. Thus, 50 ppm could the suitable concentration for MG dye for optimal operation conditions of developed nanophotocatalyst.

#### Effect of initial catalyst dosages

The change in the degradation efficiency at initial catalyst dosages of 62.5, 125, 250, 500, and 750 mg/L was studied under initial dye concentration of 50 ppm, temperature of 25 °C, contact time of 180 min, and dye pH = 4.1, and the results were illustrated in Fig. 8d. As expected, at a low dosage of catalysts, the degradation efficiency was relatively low for all samples and enhanced by increasing the catalysts dosage to 250 mg/L, which could be attributed to the increase of active sites by increasing the catalyst dosage, which provided a higher number of active radicals. The further increase of catalyst dosage than 250 mg/L has no significant effect on the degradation efficiency. This could be attributed to increasing the solution turbidity, darkness, and agglomeration of catalyst particles, which dominate light scattering phenomena, resulting in limited light absorption by the photocatalyst surface and leads to inhibition of degradation progressing [44, 88]. SiNP-MOF5 photocatalyst achieved the highest catalytic efficiency, and the photocatalytic degradation % values were found to be 91.71, 92.59, and 94.37% at catalyst dosages 250, 500, and 750 mg/L. Thus, due to the inferior change in the degradation efficiency upon huge increase in catalyst dosage, thus, the 250 mg/L could be considered as the optimum catalyst dosage form efficiency and economic point of views.

#### Effect of temperature

Figure 8e shows the effect of temperature on the degradation efficiency of MG dye for all prepared nanophotocatalyst at initial dye concentration of 50 ppm, catalyst dosage of 0.250 g/L, contact time of 180 min, and dye pH of 4.1. The results reveled that the degradation efficiency was increased upon temperature increases from 25 to 45 °C in a significant value for SiNP-MOF0 and 1, while the change was not significant in the SiNP-MOF5, and 7.5. This phenomenon could be due to the fact that the photocatalytic degradation process of organic dyes is relatively unaffected by temperature, especially when using low bandgap catalysts [88]. As a result, the changes in degradation percentage in SiNP-MOF5, and 7.5 photocatalysts that have relatively low bandgap are rather insignificant [88–90]. On the other hand, for the SiNP-MOF0 and 1 samples, because of their ability to photodegradation was less than the rest of the samples (Fig. 8a), the susceptibility of these samples to increase degradation efficiency with increasing temperature much higher than its counterparts. This is due to the solution contains large amount of MG molecules that have not yet reached the surface of the photocatalysts, thus, as the temperature rises, more MG is transferred from solution to adsorption sites on the catalyst surface, allowing more dye molecules to enter the photocatalyst matrix and interact with numerous active sites and in turn encourage and facilitate the photocatalytic degradation process [6, 91]. Additionally, as the temperature rises, the solution’s viscosity decreases, the dye molecules’ rate of diffusion into the catalyst matrix increases [6]. However, in the SiNP-MOF5, and 7.5 photocatalysts the concentration of MG molecules in the surrounding solution is not too much because they had higher photocatalytic degradation efficiency at room temperature, thus, further increase in the temperature hardly affect the degradation rate.

#### Effect of pH

The importance of pH study lies in its ability to change the charge of the photocatalyst surface and isoelectric point, which must be calculated to detect the zero-point charges to investigate the photocatalytic oxidation performance [92, 93].The effect of pH at 5 points (from pH = 3 to pH = 10) on the degradation efficiency of MG dye at initial dye concentration of 50 ppm, temperature of 25 °C, contact time of 180 min and catalyst dosage of 0.250 g L^−1^ was studied on SiNP-MOF0 and SiNP-MOF5 samples and results were presented in Fig. 8f. The MG solution pH was adjusted before the adsorption stage using 0.1 M of NaOH and HCl. The highest photocatalytic degradation efficiency was found to be ~ 69.02% for SiNP-MOF0 at pH 7 and 95.45% for SiNP-MOF5 at pH 6 (Fig. 8f). This could be attributed to the electrostatic attraction between the positive charge on the MG molecules and the photocatalyst surface that increases the diffusion of dye molecules into active sites on the catalyst, encouraging the degradation rate [92]. However, the photocatalytic degradation efficiency decreased at extremely low and extremely high pH can be explained as following, at lower pH, the functional groups on the photocatalyst surface are protonated, causing the change in the surface charge to a positive charge producing a repulsion force between the MG molecules and the photocatalyst surface which inhibits the degradation rate [94, 95]. At extremally high pH, the concentration of OH^-^ increased and formed a bond with the photocatalyst surface positive holes [88], inhibiting the degradation rate, so the rate of photocatalytic degradation decreased.

### Kinetic, isotherm, and thermodynamics study

**Fig. 9:**
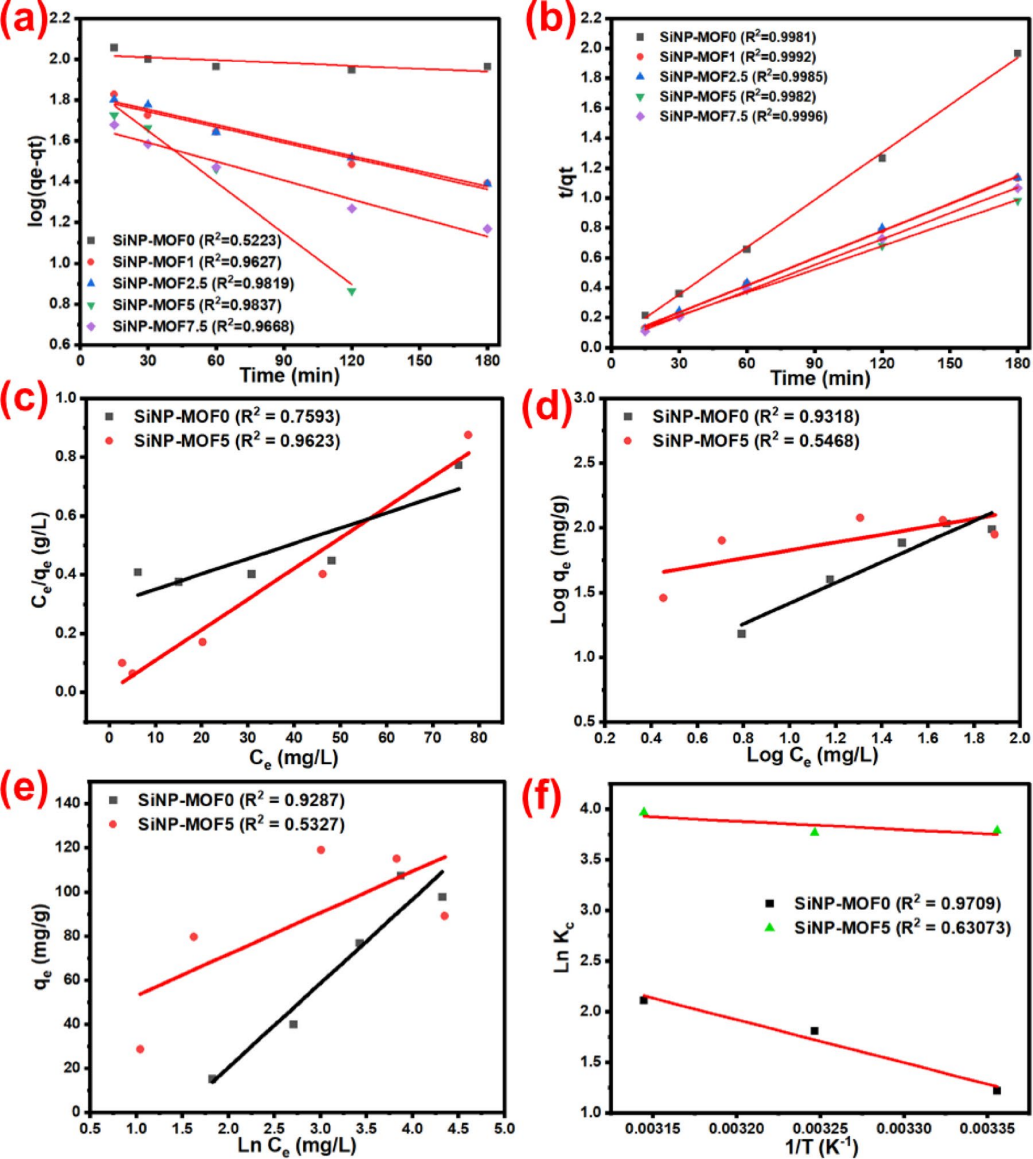
Pseudo-first-order (**a**) and pseudo-second-order (**b**), kinetic plots for all photocatalysts. Langmuir (**c**), Freundlich (**d**), and Temkin (**e**) isotherm plots for SiNP-MOF0 and SiNP-MOF5 photocatalysts. Van’t Hoff plot for the photocatalytic degradation of MG onto the SiNP-MOF0 and SiNP-MOF5 photocatalysts (**f**).

To better understand the photocatalytic degradation mechanism of MG, pseudo-first-order and pseudo-second-order kinetic models were studied. Kinetics study of photocatalytic degradation of MG dye via SiNP-MOFx photocatalysts were studied at 0.250 g/L, and the degradation rate was demonstrated by studying the contact time up to 180 min. The liner plot of Log (q_e_–q_t_) vs. t for the pseudo-first-order kinetic model and t/q_t_ vs. t for the pseudo-second-order kinetic model is shown in Fig. 9a and b, respectively. K1 and K2 are constant values of the two models, correlation coefficients (R^2^), and experimental data are shown in Table 2. For all SiNP-MOFx photocatalysts, the correlation coefficient R^2^ for the pseudo-second-order model exceeded that of the pseudo-first-order model. All R^2^ values were greater than 0.99 and very close to 1. These findings indicate that the photocatalytic degradation of MG can be accurately described by a pseudo-second-order kinetic model. On the other hand, the difference between the calculated q_e2_ calculated by the pseudo-second-order equation and the experimental q_e_ value is lower than the difference between the theoretical q_e1_ calculated by the pseudo-first-order equation and the experimental qe values. this affirming the suitability of pseudo-second-order kinetic model to describe the MG photocatalytic degradation process.

**Table 2:** Pseudo-first-order and pseudo-second-order models kinetic constants and correlation coefficient values for all prepared catalysts.

Catalyst	Pseudo first order			Pseudo second order			EXP
	q_e1 (calculated)_ (mg/g)	K_1_ (min^−1^)	R^2^	q_e2 (calculated)_ (mg/g)	K_2_ (g/mg min)	R^2^	q_e_ (mg/g)
SiNP-MOF0	105.63	0.001067	0.5223	94.70	0.002972	**0.9981**	91.52
SiNP-MOF1	66.02	0.005839	0.9627	164.90	0.000729	**0.9972**	158.82
SiNP-MOF2.5	67.60	0.005819	0.9819	164.84	0.00068	**0.9985**	158.95
SiNP-MOF 5	79.41	0.019273	0.9837	193.31	0.000462	**0.9987**	183.41
SiNP-MOF7.5	48.145	0.007071	0.9668	173.389	0.000999	**0.9996**	168.71

One of the key features of the developed photocatalysts is their high adsorption capacity before the photodegradation stage. To better understand this behavior, the Langmuir, Freundlich, and Temkin isotherm models for SiNP-MOF0 in comparison to the best-performing photocatalyst, SiNP-MOF5 were studied in terms of adsorption efficiency. Langmuir, Freundlich, and Temkin isotherm models were illustrated in Fig. 9c, d, and e, respectively, and the different isotherm constants were tabulated in Table 3. Equations 3, 4, and 5 express the linear forms of the Langmuir, Freundlich, and Temkin isotherm models, respectively [96].

3 $$\frac{{C}_{e}}{{q}_{e}}=\frac{1}{{K}_{L}{q}_{m}}+\frac{{C}_{e}}{{q}_{m}}$$

4 $$Log {q}_{e}=Log {K}_{f}+\frac{1}{n}Log {C}_{e}$$

5 $${C}_{e}=B Ln {A}_{t}+B Ln {C}_{e}$$

where C_e_ is the adsorption at equilibrium, K_L_ (L/mg) is the Langmuir constant and q_m_ (mg/g) is the maximum capacity of the adsorption, K_F_(L/g) and n are the Freundlich isotherm constants, respectively. From the results, we can conclude that, for SiNP-MOF5, Langmuir isotherm was suitable to describe the experimental data better than the other two models, and the correlation coefficient value (R^2^) was found to be 0.9623 (Table 3). This confirms the monolayer adsorption of MG dye molecules onto the SiNP-MOF5 surface. However, for SiNP-MOF0, in contrast to the Langmuir and Temkin models, the Freundlich isotherm was able to better reflect the experimental results for the SiNP-MOF0 sample, with a correlation coefficient value (R^2^) of 0.9318 (Fig. 9d). This confirms the multilayer adsorption on SiNP-MOF0 surface and predicts the positive or neutral effect of the increasing MG dye concentration, and this could explain the different behavior of the SiNP-MOF0 sample, in contrast to the rest of the samples containing Si-NPs.

**Table 3:** Adsorption isotherm constant for MG adsorption by the SiNP-MOF0 and SiNP-MOF5 samples during the adsorption stage.

Catalyst	Langmuir			Freundlich			Temkin		
	K_L_ (L/mg)	q_m_ (mg/g)	R^2^	K_F_ (L/mg)	n	R^2^	B (J/mol)	A_*T*_* (L/g)*	R^2^
SiNP-MOF0	0.017	192.4	0.7593	4.17	1.26	**0.9318**	0.026	1.66E-56	0.9287
SiNP-MOF5	2.08	96.05	**0.9623**	33.24	3.28	0.5468	0.05	1.41E + 34	0.5327

The thermodynamic parameters of the fabricated photocatalysts during the photocatalytic degradation process was evaluated by monitoring the degradation of the MG at different temperatures (25°C to 45°C). Standard enthalpy (∆H), Gibbs free energy (∆G), and standard entropy (∆S) were studied for SiNP-MOF0 and SiNP-MPF5 photocatalysts and data were shown in Fig. 9f and tabulated in Table 4. The correlation coefficient values (R^2^) were 0.9709 and 0.6307 for SiNP-MOF0 and SiNP-MOF5, respectively, which confirms the previous conclusion for the significant effect of temperature on the degradation efficiency of SiNP-MOF0. However, inferior effect for SiNP-MOF5 which displays low R^2^ value (Table 4). Moreover, the negative values of ∆G reveal the spontaneity of the degradation process [7]. The increase in the − ∆G value by increasing the temperature from 25°C to 45°C, especially in SiNP-MOF0, confirms the previous finding for the favorability of the MG dye to diffuse into the catalyst matrix at high temperatures and in turn facilitate the degradation process. The fact that the ∆H value was greater than zero indicated that MG degradation was endothermic, particularly in SiNP-MOF0. This means that a higher temperature will allow MG to diffuse onto the catalyst framework, offering easier connection between the dye molecules and the active sites on SiNP-MOF0 [97].

**Table 4:** The change in thermodynamic parameters (ΔH°, ΔS°, and ΔG°) and R^2^ from Van’t Hoff plot for the degradation of MG dye via SiNP-MOF0 and SiNP-MOF5 samples.

Catalyst	ΔH° (KJ/mol)	ΔS° (KJ/K mol)	− ΔG° (KJ/mol)			R^2^
			298 K	308 K	318 K	
SiNP-MOF0	35.259	0.129	− 3.013	− 4.638	− 5.574	0.9709
SiNP-MOF5	6.975	0.055	− 9.389	− 9.640	− 10.494	0.63073

### Radical trapping experiment and photocatalytic degradation mechanism

**Fig. 10:**
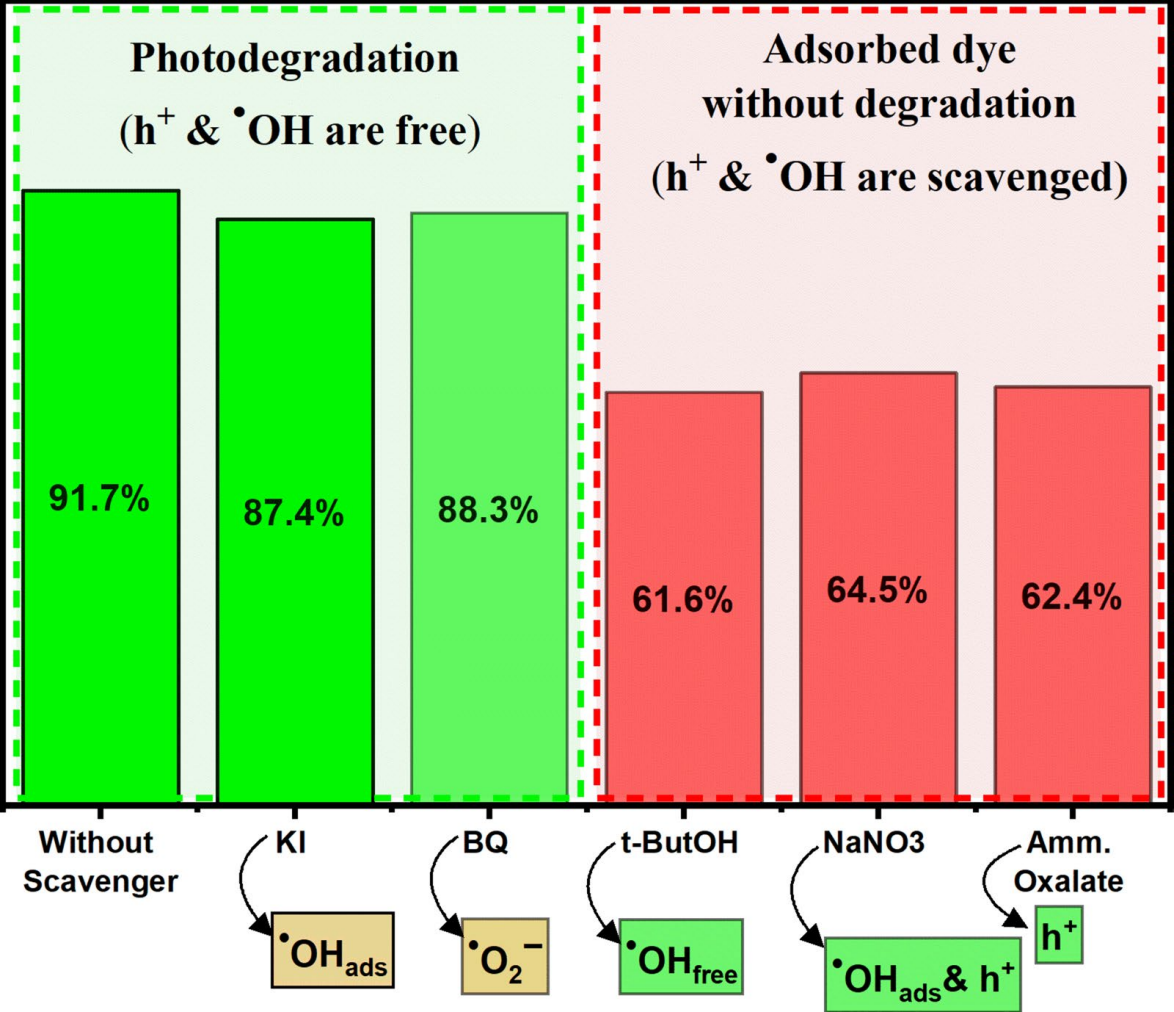
Photocatalytic degradation of MG dye in the presence of different scavengers using SiNP-MOF5 photocatalyst.

Photocatalytic degradation of MG dye was studied in the presence of different quenchers to evaluate the reactive species responsible for the photodegradation effect. Thus, the radical trapping experiment was studied at initial MG concentration of 50 ppm, temperature of 25 °C, contact time of 180 min, catalyst dosage of 0.250 g/L, dye pH, and in the presence of targeted quencher using SiNP-MOF5 photocatalyst. 3 mM of five different quenchers was added individually to the reaction system for scavenging the active radicals. Thus, KI was applied to trapping the ^·^OH_free_ and ^·^OH_ads_, t-Butyl alcohol served as ^·^OH_free_ quencher, NaNO_3_ act as ^·^OH and h^+^ scavenger. However, ammonium oxalate inhibitor for h^+^, and Benzoquinone for ^·^O_2_^−^ trapping. As shown in Fig. 10 the photocatalytic degradation of MG dye was dramatically decreased in presence of t-ButOH, NaNO_3_, and Amm. Oxalate which response for ^·^OH_free_, ^·^OH & h^+^, and h^+^, respectively. The percentage of dye removed detected were 61.6, 64.5, 62.4% for t-ButOH, NaNO_3_, and Amm. Oxalate, respectively, these removal efficiencies were almost achieved during the adsorption stage only (first 30 min in dark) without any influence in photoirradiation stage (3 h light). It is worth noting that the adsorption capacity of the SiNP-MOF5 sample during the adsorption stage under the same conditions was 59.5%, as descried earlier (Fig. 8a and b). The attained data revealed that the photocatalytic degradation process of MG was completely suppressed in the presence of these scavengers, which predict the main active radicals responsible for the photocatalytic degradation of MG are ^·^OH & h^+^ radicals.

The photocatalytic degradation mechanism is determined based on data originated from various characterization analyses, particularly UV–Vis and XPS, in conjunction with radical trapping experiments. The results of the bandgap engineering indicate that the surface decoration of Ni-MOF (3.97 eV) by 5% of Si-NPs reduces the bandgap of new developed SiNP-MOF5 to 2.68 eV. According to the XPS data, the valence band for Si-NPs, SiNP-MOF0, and SiNP-MOF5, were set at 0.62, 1.97, and 1.52 eV (Fig. 6g–i), respectively. Also, the conduction band was calculated to be at − 0.5, − 2, and − 1.16 eV for Si-NPs, SiNP-MOF0, and SiNP-MOF5, respectively.

**Scheme 2:**
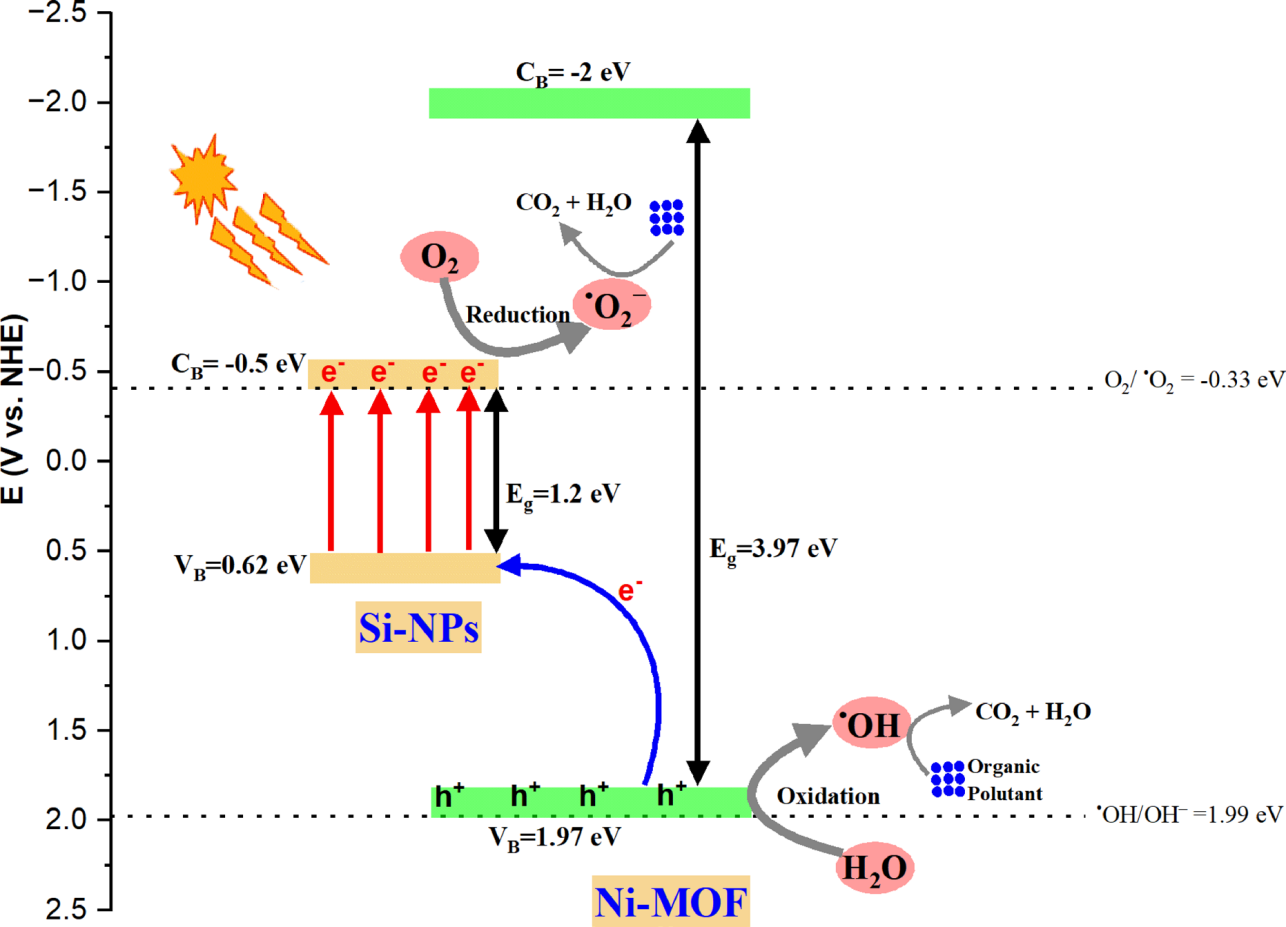
A plausible mechanism for organic pollutant degradation via Si-NPs/Ni-MOF heterojunction under visible light.

Therefore, based on the previous results, the photocatalytic degradation mechanism can be predicted as presented in Scheme 2 for the photodegradation of various organic pollutants under visible light using the Si/Ni-MOF heterojunction photocatalyst. Hence, according the finding in this study and relevant literature [37, 40, 41, 98–100], it is obvious that Ni-MOF is a promising photocatalyst in the decomposition of organic pollutant because its stability and relatively high surface area, but it lacks to proper band gap to work in visible light. Therefore, the reported literature focused on using it in photodegradation under UV light irradiation [35]. Accordingly, it is thought that in the Si/Ni-MOF heterojunction, Ni-MOF serves as the primary photocatalyst, while Si serves as a sensitizer by absorbing visible light. Hence, under visible-light illumination, the electrons in the V_B_ level of Si are excited and transfer to its C_B_ leaving the h^+^ behind on the V_B_. Then, the electrons in the V_B_ of Ni-MOF can move to the Si V_B_ because the V_B_ of Si sensitizer is very close to that of Ni-MOF, generating h^+^ on the MOF V_B_; consequently, photocatalytic oxidation reactions are initiated as presented in scheme 2. The positive holes (h^+^) in the MOF V_B_ can be scavenged by OH^–^ or the adsorbed water (H_2_O) on the surface of the catalyst, generating the reactive hydroxyl radicals (^·^OH) in aqueous media as presented in scheme 2 and described in Eqs. (6) and (7) [101]. On the other hand, the exited electrons on the C_B_ of Si initiate various reduction reactions including formation of ^·^O_2_ as shown in Eq. (8).

6 $$\left( {{\text{OH}}^{-} } \right)_{{{\text{ads}}}} + {\text{h}}^{ + } \to^{ \cdot } {\text{OH}}$$

7 $$\left( {{\text{H}}_{2} {\text{O}}} \right)_{{{\text{ads}}}} + {\text{h}}^{ + } \to {\text{H}}^{ + } +^{ \cdot } {\text{OH}}$$

8 $${\text{O}}_{2} + {\text{e}}^{ - } \to^{ \cdot } {\text{O}}_{2}^{ - }$$

As a result of the strong oxidative potential of the holes created in the Ni-MOF’s V_B_, and reductive potential of superoxides formed on the C_B_ of Si, efficient and complete degradation of organic molecules could be achieved to form CO_2_ and H_2_O.

### Photocatalyst reusability and economic feasibility study

**Fig. 11:**
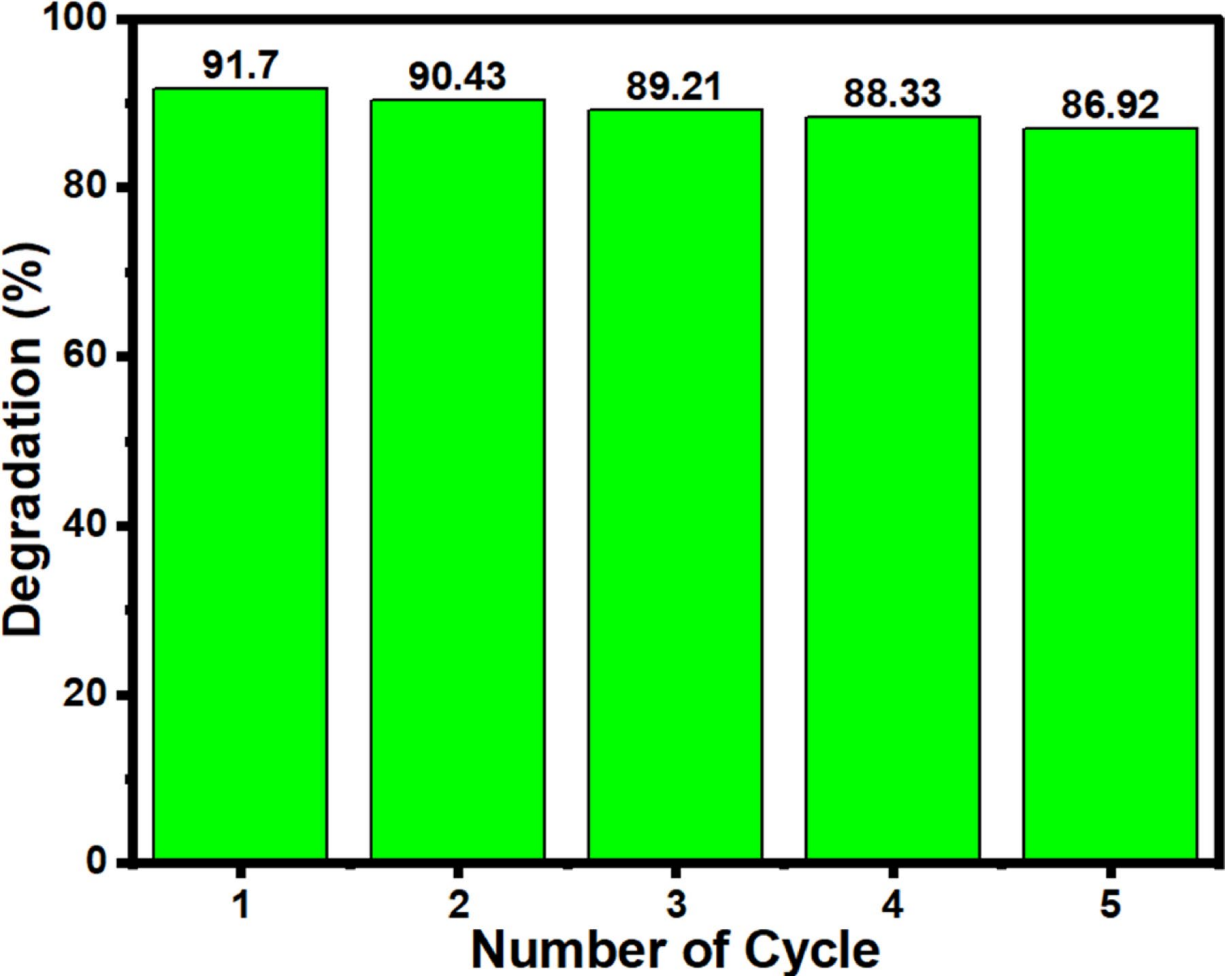
Reusability study for five cycles for SiNP-MOF5 sample.

A study of recyclability is one of the most important parameters when evaluating economic feasibility for industrial applications. To assess the recyclability of SiNP-MOF5, it was recovered via centrifugation at 15,000 rpm after each cycle of MG degradation. The recovered photocatalyst was repeatedly washed with ethanol and dried before reusing in a new photodegradation cycle. The reusability was studied at initial MG concentration of 50 ppm, temperature of 25 °C, contact time of 180 min, catalyst dosage of 0.250 g/L, and pH of 4. Remarkably, as shown in Fig. 11, the photocatalytic efficiency of SiNP-MOF5 was not significantly affected even after five cycles of reusing, confirming its stability and ability to reuse as an efficient photocatalyst.

On the other hand, to accurately determine the feasibility of using the prepared SiNP-MOF5 photocatalyst for wastewater treatment, the financial cost of the developed SiNP-MOF5 photocatalyst was evaluated. As mentioned above, 0.25 g of SiNP-MOF5 is sufficient to treat 1 L of wastewater within 3 h. Therefore, based on this finding, the cleaning of 4 m^3^ of wastewater need 1 kg of Si-NP-MOF5. Regardless of the capital costs, the cost of production of 1 kg of the SiNP-MOF5 photocatalyst was conducted based on Table S2. Thus, the production of 1 kg of SiNP-MOF5 will be cost 154.5 $ which is enough to clean 4 m^3^ wastewater to be suitable for agriculture purpose as indicated below. Interestingly, according to reusability study conducted for Si-NP-MOF which revealed that it could be reused at least 5 times efficiently. This means that 1 kg of SiNP-MOF5 photocatalyst will be sufficient to treat at least 20 m^3^ of wastewater, thereby confirming the economic feasibility of the photocatalyst developed in this study.

### Evaluation of the biological phytotoxicity and antimicrobial activity

**Fig. 12:**
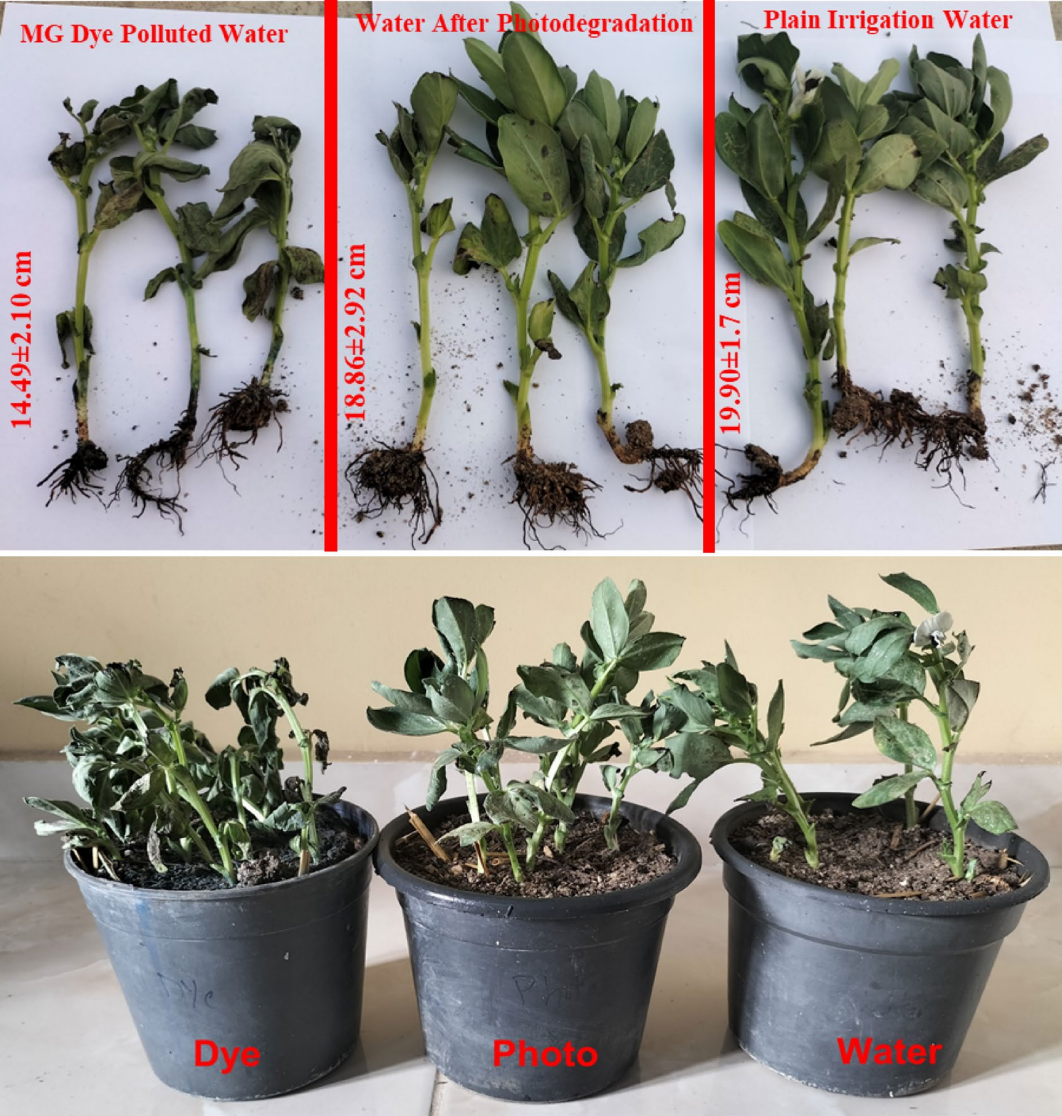
Experiment for germinating faba bean plants using three types of water: regular irrigation water, water contaminated with MG dye, and the same contaminated water after the photodegradation process using the SiNP-MOF5 photocatalyst.

To assess the biological phytotoxicity of the water that has been photocatalytically degraded using SiNP-MOF5 photocatalyst, faba bean plants were grown in three types of water; plain irrigation water, water polluted with MG dye, and the same polluted water after the photocatalytic degradation of MG dye using SiNP-MOF5. The experimental design for assessing biological phytotoxicity, including the germination and implantation procedures, is described in detail in the Supplementary Data (Fig. S7). The faba bean plant was selected due to its importance as a source of plant protein and the increasing demand in the global market, in addition to its fast growth rate [102, 103]. The planting period was 30 days, with regular irrigation to obtain well-developed root and shoot systems. Finally, the plants were pulled out in order to measure some parameters like, plant height, degree of vitality of the leaves and stems, and influence of the type of irrigation water on these parameters. As shown in Fig. 12, the mean of plant length was 19.90 ± 1.7, 18.86 ± 2.92, and 14.49 ± 2.10 cm for plants that irrigated with clean water, photodegraded water, and dye polluted water, respectively. The plants irrigated regularly with water contaminated with the dye exhibited relative stunting and wilting in their vegetative growth, indicating the toxicity of the contaminated water and its hindrance to normal plant growth. No significant differences were observed between the plants irrigated with regular water and those irrigated with water treated with SiNP-MOF5 photocatalyst, suggesting a reduction in the biological phytotoxicity of the dye-contaminated water after treatment. This confirms the efficiency of SiNP-MOF5 as a photocatalyst and the suitability of the treated water for agricultural purposes.

**Fig. 13:**
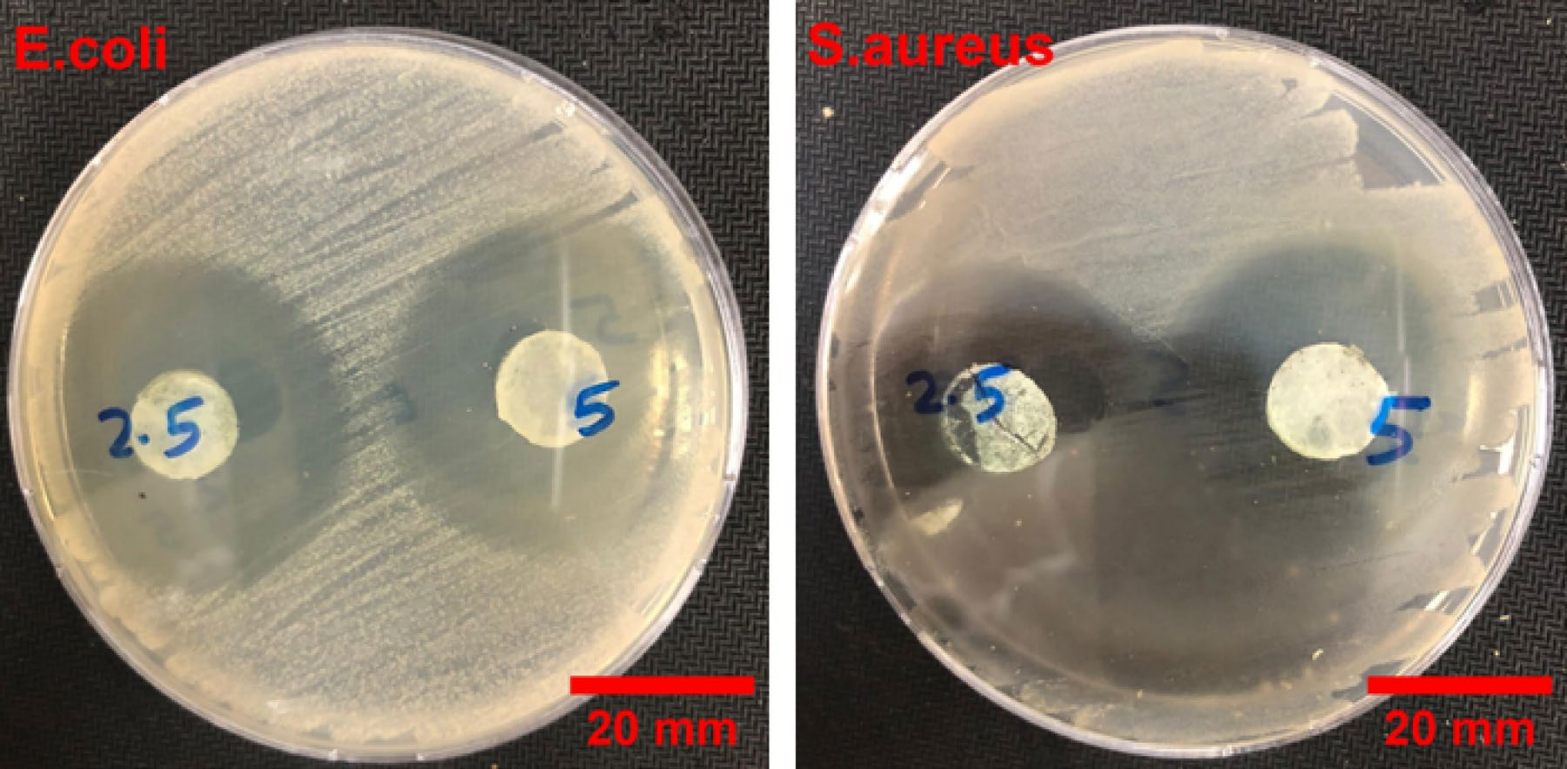
Antibacterial activity and Inhibition zone against *S.aureus* and *E. coli* by SiNP-MOF2.5 and SiNP-MOF5.

The antibacterial activities of the prepared SiNP-MOF2.5 and 5 were obtained from the disc diffusion method against *S. aureus and E. coli* (Fig. 13). The antibacterial clear inhibition zone of SiNP-MOF2.5 and 5 against *S. aureus* were found to be 47.7 mm, and 28.3 mm, respectively, and against the *E. coli* were found to be 30 mm and 29 mm, respectively. These results displayed that developed nanophotocatalysts have inhibitory actions against the growth of different bacterial strains (Fig. 13).

#### Comparative study

It is important to compare the efficiency of the developed nanophotocatalyst with the previously reported photocatalyst in photocatalytic degradation of various organic pollutants. Table 5 illustrates the comparison of degradation efficiency of SiNP-MOF5 against various reported photocatalysts. It can be concluded that incorporating Si-NPs on the surface Ni-MOF nanosheets offer smart bandgap engineering and in turn resulted in the fabrication of a highly effective photocatalyst. Which capable of degrading MG, CV, and TC within 180 min using a catalyst dosage of 0.250 g/L under visible light and achieving superior photocatalytic degradation efficiency rather than various reported systems [37, 40–44, 104, 105].

**Table 5:** Comparison of the photodegradation efficiency of SiNP-MOF5 photocatalyst with previously reported studies.

No	Photocatalyst	Pollutant	Light source	Cat. dosage (g/L)	Pollutant Conc. (mg/L)	Time (min)	Eff. (%)	References
1	Ni-MOF/BiOBr	Methylene Blue Ciprofloxacin	300W Xenon lamp	0.3 0.3	20 20	120 120	82.8 92.8	[40]
2	Ni-MOF/Bi_2_WO_6_	Methylene Blue	300W Xenon lamp	0.5	20	60	98.8	[37]
3	BiOCl/Ni-MOF-74	Tetracycline Rhodamine B	300W Xenon lamp	0.4 0.4	25 30	100	93 97	[41]
4	Ni-MOF Co/Ni-MOF /BiOI-15%	Methylene Blue	Sunlight	0.08	10	240	18 99	[42]
5	ZnFe_2_O_4_@Co/Ni-MOF	Congo red	50 W LED lamp	1	10	75	98	[43]
6	RhB/MIL-125 (Ti)	Methyl Orang	300W Xenon lamp	0.710	20	60	90	[104]
7	BiVO_4_/MIL-125(Ti)	Rhodamine B	500W Xenon lamp	0.5	10	180	92	[105]
8	Dual Z scheme NiS/ZrO_2_/CdS	Tetracycline Sulfamethoxazole Cephalexin Ofloxacin Amoxicillin Doxycycline	Sunlight	0.5	50 30 30 50 30 30	120	97 75 83 73 88 98	[44]
9	**SiNP-MOF5**	**Malachite Green** **Crystal violet** **Tetracycline**	**100 W LED lamp**	**0.25**	**50** **10** **10**	**180**	**91.7 86.8** **95.2**	**This work**

## Conclusion

A novel, efficient and cost-effective heterojunction nanophotocatalysts based on green impregnation of Si-NPs onto the 2D Ni-MOF nanosheets were fabricated. The Si-NPs act as sensitizers for Ni-MOF nanosheets decreasing the band gap from 3.97 to 2.68 eV encouraging the photocatalytic degradation under the visible light. The developed photocatalysts, especially SiNP-MOF5, achieved high activity in photocatalytic degradation of various organic pollutants, including MG, CV, and TC, achieving degradation efficiencies of 91.7%, 86.8%, and 95.2%, respectively, at an initial dye concentration of 50, 10, and 10 ppm, respectively, and the catalyst dose of 0.25 g/L of high-performance photocatalyst SiNP-MOF5, within 180 min under visible light. The great advantage of the developed photocatalyst is that they are based on the preparation of Si-NPs from abundantly available and economical sand offering large-scale production. The mechanism of photodegradation confirmed the ^·^OH & h^+^ are the main active radicals responsible for the photodegradation process. Additionally, the reusability and economic feasibility studies indicates that the developed catalyst can be reused efficiently for up to 5 cycles and the cost for cleaning of approximately 20 m^3^ of wastewater efficiently, is about $154.50. The biological phytotoxicity study shows no significant differences between the plants irrigated with regular water and those irrigated with water treated with the developed photocatalyst. Also, the developed photocatalysts displayed excellent inhibition against different bacterial strains growth and in turn afford excellent antibacterial properties for wastewater.

## Supplementary Information

Below is the link to the electronic supplementary material.

Supplementary Material 1

## Data Availability

All data generated or analyzed during this study are included in this published article and its supplementary information files.
