# Supplementary material for: Impact of decoration of spherical silicon nanoparticles on 2D Ni-MOF nanosheets for integrating superior photodegradation of toxic organic pollutants in aqueous solution
Source: Sci Rep. 2025 Sep 12;15:32447. doi: 10.1038/s41598-025-17952-9 (PMC12432178; doi:10.1038/s41598-025-17952-9)
Supplement: Supplementary file 1 — Supplementary Material 1 [file 41598_2025_17952_MOESM1_ESM.docx]

**Impact of Decoration of Spherical Silicon Nanoparticles on 2D Ni-MOF Nanosheets for Integrating Superior Photodegradation of Toxic Organic Pollutants in Aqueous Solution**

Amin M. Elkony ^a^, Hosni A. Gomaa ^a^, Ahmed A. Omran ^a^, Nour F. Attia ^b*^

^a^ Department of Chemistry, Faculty of Science, Al-Azhar University, Nasr City, Cairo 11884, Egypt

^b^ Gas Analysis and Fire Safety Laboratory, Chemistry Division, National Institute for Standards, 136, Giza 12211, Egypt

*Corresponding author

E-mail addresses: [drnour2005@yahoo.com](mailto:drnour2005@yahoo.com)


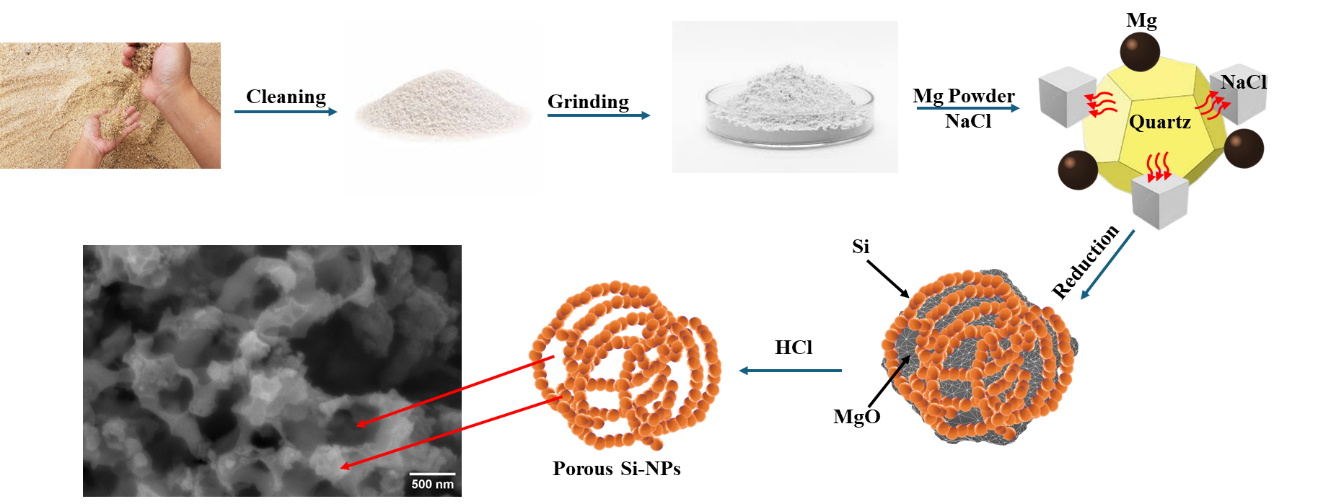


**Scheme S1.** Schematic diagram shows the steps of synthesis of Si-NPs via magnesiothermic reduction method.


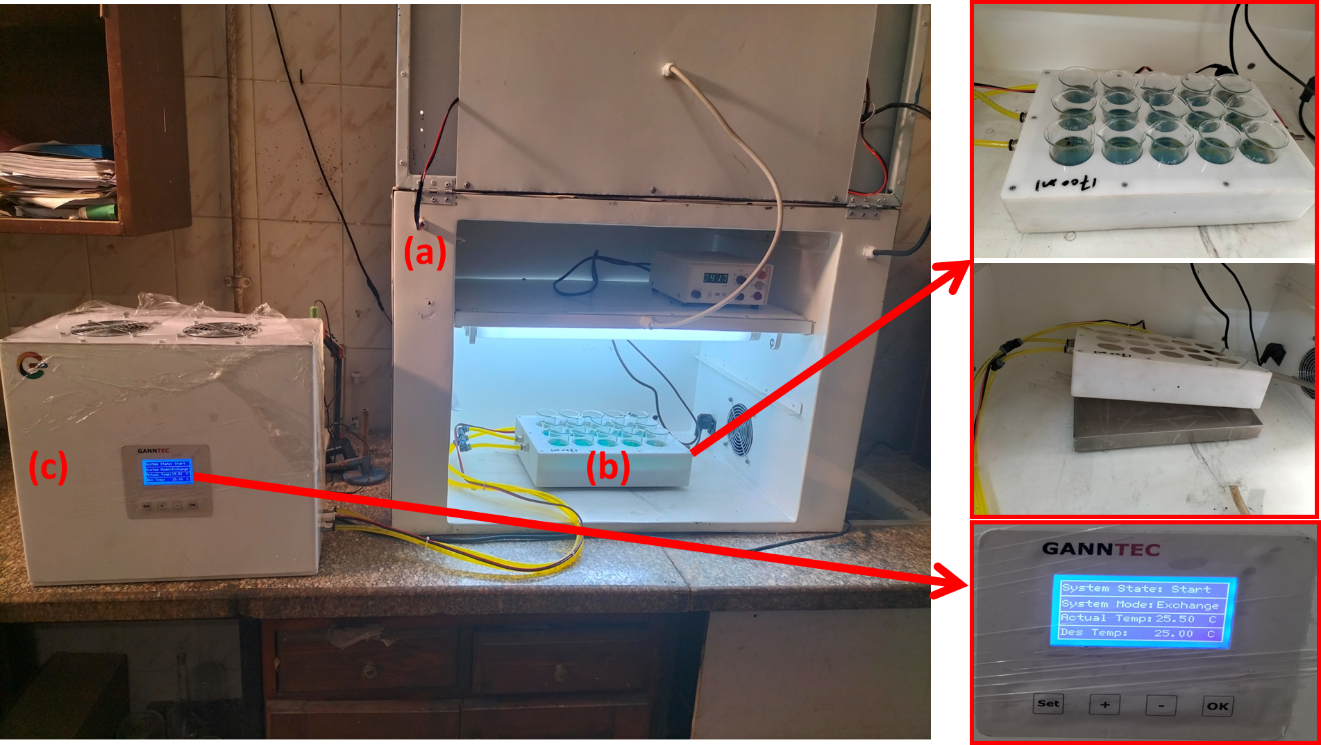


**Fig. S1.** A photograph of the photocatalytic degradation system including the light box (a), multi stirrer (15*1) caped with water heat exchanger unit (b), and the temperature regulator unit (c).

**
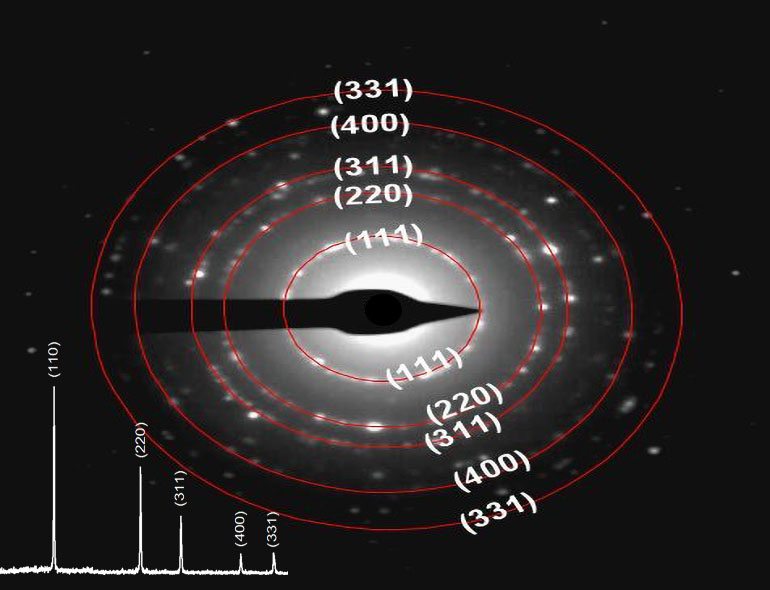
**

**Fig. S2.** Selected area electron diffraction pattern of Si-NPs.


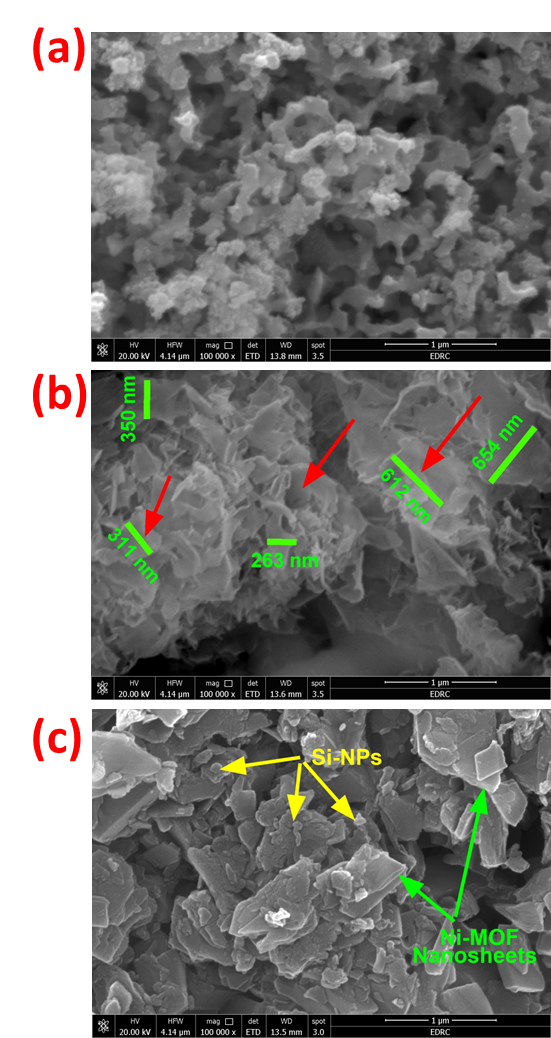


**Fig. S3.** SEM images at a magnification of 100,000X of Si-NPs (a), SiNP-MOF0 (b), and SiNP-MOF5 (c) samples.


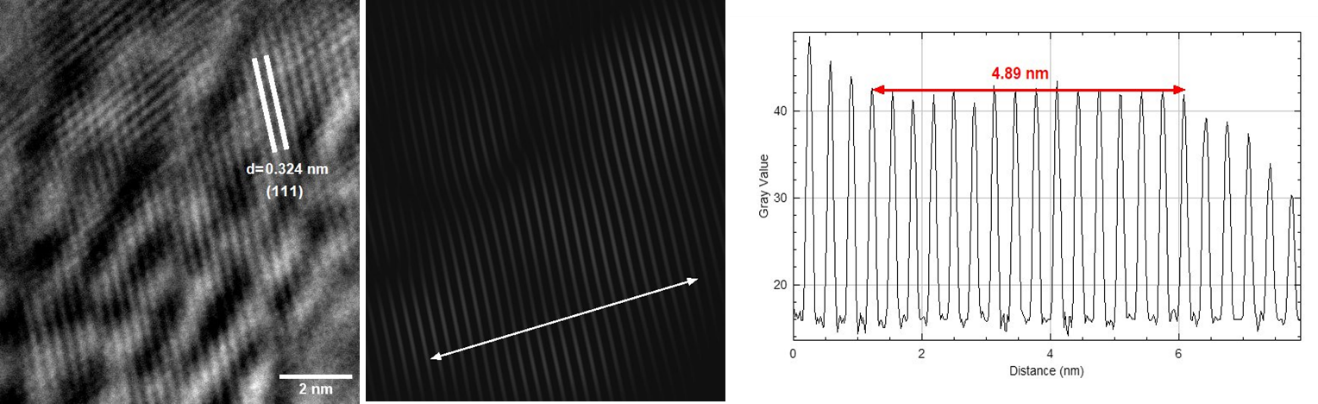


**Fig. S4.** Image analysis results of interplanar distance measurements.

**Table S1.** illustrates the BET surface areas and pore properties of sample Si-NPs, SiNP-MOF0, and SiNP-MOF5.

| Sample | BET surface areas (m^2^ g^−1^) | Pore volume (cm^3^ g^−1^) |
| --- | --- | --- |
| Si-NPs | 31.02 | 0.1937 |
| SiNP-MOF0 | 17.221 | 0.2276 |
| SiNP-MOF5 | 5.801 | 0.0869 |
|  |  |  |

**
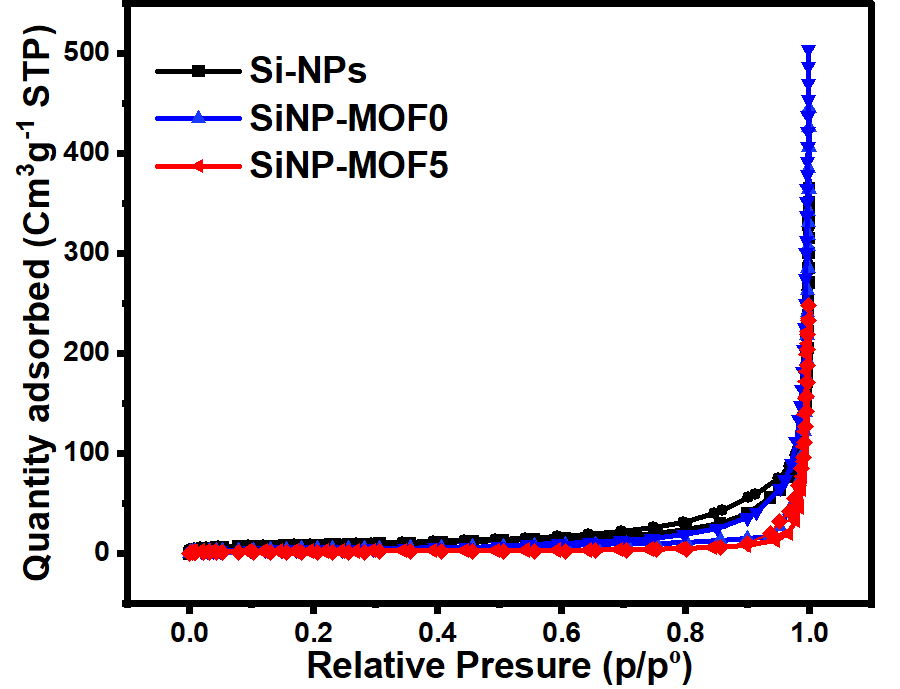
**

**Fig. S5.** N_2_ adsorption-desorption for samples Si-NPs, SiNP-MOF0, and SiNP-MOF5.


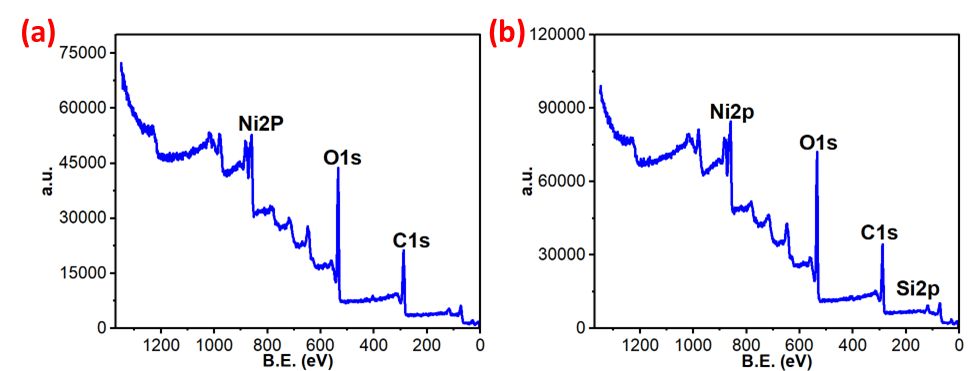


**Fig. S6**. XPS survey scan spectrum of SiNP-MOF0 (a), SiNP-MOF5 (b).

**Table S2.** Economic evaluation and Cost-benefit analysis for producing 1 Kg of SiNP-MOF5 photocatalyst.

| Component | Materials | Required Quantity for Producing 1 Kg of SiNP-MOF5 | Cost  ($, US) | Total Cost  ($, US) |
| --- | --- | --- | --- | --- |
| Ni-MOF (950 g) | Ni(NO₃)₂·6H₂O  H₂BDC  DMF  EtOH | 0.63 Kg  0.56 Kg  19.9 L  1.4 L | 39.60  18.94  57.1  2.81 | 118.45 |
| Si-NPs (50 g) | Sand  Mg Powder  NaCl  HCl | 0.125 Kg  0.1 Kg  0.125 Kg  0.5 L | 0.02  9.90  0.10  0.99 | 11.01 |
| Operating Cost | Mechanical and electrical costs |  | - | 25 |
| **Overall Cost** | - | - | - | **154.5** |

**Experimental Design for Assessing Biological Phytotoxicity**

To evaluate the phytotoxicity of the treated water, a controlled lab-scale experiment was conducted (not expanded to large-scale field trials) with the following steps:

1. **Seed Source**: Faba bean seeds were purchased from a local Egyptian market, which is the primary source for most farmers in the region.
2. **Germination**: Seeds were soaked in three water types (normal irrigation water, MG-contaminated water [50 ppm], and post-photocatalytically treated MG water) and germinated in sealed lab containers to ensure sterility (Fig. S7)
3. **Soil Preparation**: Identical soil conditions were maintained across three cultivation pots. Pre-germinated seeds were transplanted into these pots and irrigated with their respective water types.
4. **Planting Conditions**: Cultivation began on **1st November** (the optimal planting period for faba beans in Egypt).
5. **Growth Maintenance**: All pots received equal sunlight exposure and were irrigated every 5 days to prevent drought stress (Fig. 12).
6. **Harvesting**: After 30 days, plants were carefully uprooted to measure key parameters: **plant height**, **leaf/stem vitality**, and the influence of water type on these traits (Fig. 12).
7. **Focus of Study**: The experiment targeted only the water’s impact on germination rate, shoot elongation, and leaf vitality to assess the **biological phytotoxicity** of treated water.


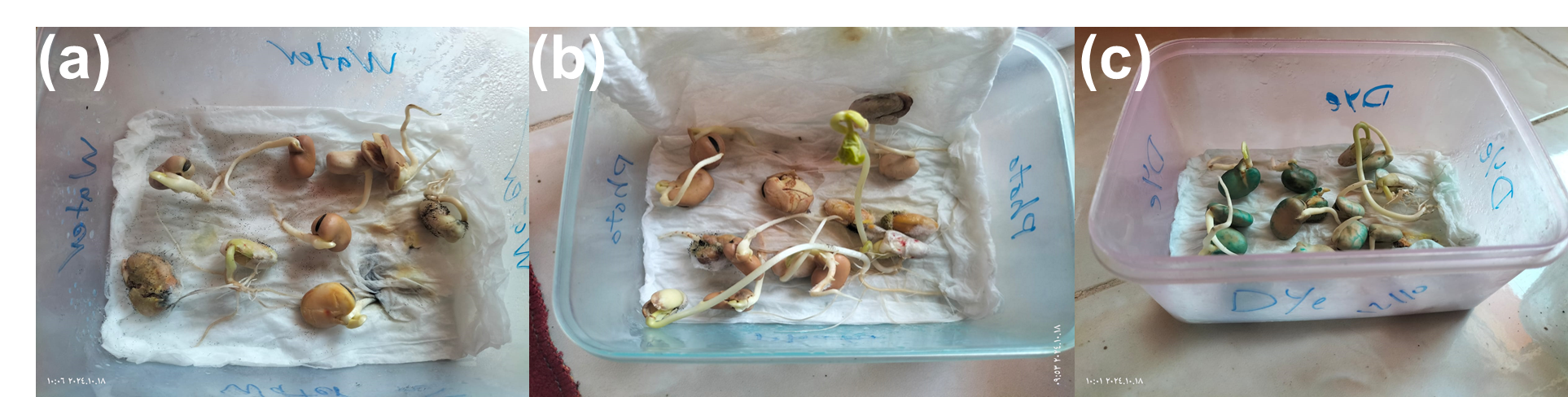


**Fig. S7.** Digital phots of germination of seeds soaked in three water types (a) normal irrigation water, (b), post-photocatalytically treated MG water (50 ppm) and (c) MG-contaminated water [50 ppm] and germinated in sealed lab containers to ensure sterility.
